# Utility of in vivo metabolomics to support read-across for UVCB substances under REACH

**DOI:** 10.1007/s00204-023-03638-6

**Published:** 2024-01-24

**Authors:** H. Kamp, N. Aygun Kocabas, F. Faulhammer, N. Synhaeve, E. Rushton, B. Flick, V. Giri, S. Sperber, L. G. Higgins, M. G. Penman, B. van Ravenzwaay, M. Rooseboom

**Affiliations:** 1 BASF Metabolome Solutions GmbH, Berlin, Germany; 2 TotalEnergies Refining & Chemicals, Seneffe, Belgium; 3 Shell Global Solution International B.V, The Hague, The Netherlands; 4 BASF SE, Ludwigshafen, Germany; 5 ExxonMobil, Machelen, Belgium; 6 LyondellBasell, Rotterdam, The Netherlands; 7 Environmental Sciences Consulting, Altrip, Germany; 8 LOA C/O Penman Consulting Ltd, Brussels, Belgium; 9 NUVISAN ICB GmbH, Toxicology, 13353 Berlin, Germany

**Keywords:** UVCB, Read-across, Metabolomics, REACH, New approach methodologies (NAMs), Chemical grouping

## Abstract

Structure-based grouping of chemicals for targeted testing and read-across is an efficient way to reduce resources and animal usage. For substances of unknown or variable composition, complex reaction products, or biological materials (UVCBs), structure-based grouping is virtually impossible. Biology-based approaches such as metabolomics could provide a solution. Here, 15 steam-cracked distillates, registered in the EU through the Lower Olefins Aromatics Reach Consortium (LOA), as well as six of the major substance constituents, were tested in a 14-day rat oral gavage study, in line with the fundamental elements of the OECD 407 guideline, in combination with plasma metabolomics. Beyond signs of clinical toxicity, reduced body weight (gain), and food consumption, pathological investigations demonstrated the liver, thyroid, kidneys (males only), and hematological system to be the target organs. These targets were confirmed by metabolome pattern recognition, with no additional targets being identified. While classical toxicological parameters did not allow for a clear distinction between the substances, univariate and multivariate statistical analysis of the respective metabolomes allowed for the identification of several subclusters of biologically most similar substances. These groups were partly associated with the dominant (> 50%) constituents of these UVCBs, i.e., indene and dicyclopentadiene. Despite minor differences in clustering results based on the two statistical analyses, a proposal can be made for the grouping of these UVCBs. Both analyses correctly clustered the chemically most similar compounds, increasing the confidence that this biological approach may provide a solution for the grouping of UVCBs.

**Supplementary Information:**

The online version contains supplementary material available at 10.1007/s00204-023-03638-6.

## Introduction

In the absence of validated and accepted new approach methods (NAMs) for repeated dose studies of systemic and reproduction toxicity, grouping of chemicals and subsequent read-across from data-rich chemicals belonging to the same group has been the most efficient way to support the required safety information, while keeping the number of animal testing to an absolute minimum (ECHA 2014). Read-across involves the use of relevant information from analogous substance(s) (the “source” information) to predict properties for the “target” substance(s). It needs to be ensured “…that the prediction of a property based on read-across is reliable, can be used for risk assessment and/or classification and labelling, and complies in general with the provisions in REACH for the substance under consideration according to the RAAF” (ECHA 2017a). The quality and hence confidence in the selection of the right compounds to form such a group are key, and consequently submitted read-across cases are evaluated by ECHA in a stepwise procedure using different scenarios and predefined criteria. As the quality of each individual step in this evaluation process needs to be sufficiently convincing, the threshold for acceptance of read-across is high. Challenges and considerations for “Good Read-Across Practice” (GRAP) have been presented (Ball 2016).

Chemical grouping is also used to form groups/sets of substantially similar compounds and to test within this group the most relevant ones as representatives for the group. This selection can, for example, be based on the extremes of the group with respect to chemical structure or expected toxicity to sufficiently cover the range within the group. In both cases, however, the essential component of chemical grouping is structural similarity. This results in an apparently unsurmountable obstacle when dealing with chemical entities called substances of unknown or variable composition, complex reaction products, or biological materials (UVCB), which in their very nature are not chemically well-defined mono-constituents. Such mixtures of substances are also referred to as more than one constituent substance (MOCS).

Using ECHA’s description (ECHA 2017c), UVCBs are substances that cannot be sufficiently identified because:

· The number of constituents is relatively large, and/or.
· The composition is, to a significant part, unknown, and/or.
· The variability of composition is relatively large or poorly predictable.

As a result of compositional variability, the identification of UVCB substances is mostly based on their generic description. The production source, process of formation, composition fingerprints, and physicochemical properties are used to identify UVCB substances. In an extension of the RAAF for UVCBs, ECHA provides two concepts on how to address chemical grouping for such compounds (ECHA 2017b):

- (1) In a *constituent-based approach,* the source data come from test results obtained with the individual constituents of the target substance. If a prediction is attempted for a multi-constituent substance or a UVCB target substance based on the properties of individual constituents, then the composition and concentrations of the constituents in the target substance must be accounted for in the justification for the prediction. It is noted, however, that “the test results of the individual constituents alone will have no information on possible interactions of these constituents when combined exposure to these constituents occurs.”
- (2) In a *substance-based approach,* the source data come from test results obtained with the source substance (a multi-constituent substance or a UVCB). The results are used to predict the properties of a target substance. It is acknowledged that “all possible toxicokinetic and toxicodynamic interactions among the source substances constituents are inherently reflected in the test result.” The test result does not discriminate the specific contribution of the individual constituents in the type of effect(s) observed or on their individual potency or on their possible interaction on the toxicokinetic and/or toxicodynamic level. Therefore, if a prediction is attempted for a multi-constituent substance or UVCB based on test results obtained with a source substance containing more than one main constituent, the composition and concentrations of the constituents of the target and source substances must be considered in the justification for the prediction.

In other words, the constituent-based approach is general and would be suitable for any combination of data-rich individual chemicals, at the price of not knowing exactly how they interact. The substance-based approach is specific and considers the interactions of the individual components. It requires that the source compounds have a sufficiently similar composition, implying that the concentrations of the individual constituents are known and would also be similar, which is particularly difficult for UVCBs. Consequently, there is a significant risk that the process of chemical grouping and read-across will not work sufficiently well to provide regulators with data that fulfill the requirements, resulting in undesirable animal testing and reluctance of registrants to pursue such a route given the uncertain outcome in the regulatory context.

An economically and volumetrically important set of UVCBs are resin oils and cyclic dienes. These UVCBs are produced by a similar manufacturing process involving the distillation of products from a steam cracking process. A pyrolysis gas or naphtha as starting material is steam-cracked at high temperature (800–1000 ℃) and then distilled or filtered progressively at lower temperatures (approx. > 200 ℃) to remove low carbon number fractions (typically below C9). Consequently, the substances produced have many of the same constituents, with the proportions of these constituents depending on individual producer-specific manufacturing process adjustments. The major groups of constituents in the substances within the category are indicated in Table 1. This process produces a set of similar, yet not identical UVCB substances. All the substances in the category are complex UVCBs typically containing %(w/w) ≤ 1% paraffins and/or ≤ 25% isoparaffins and/or ≤ 10% naphthenics. The category also contains circa 25% olefins (range ≤ 100%) and aromatics (circa 50%, range < 100%). In addition, the manufacturing process produces substances that may also contain above > 0.1%– ≤ 3% benzene (typically < 0.1%) and variable concentrations of dicyclopentadine (DCPD) (above 0.6%), naphthalene, toluene, xylene, and styrene. Owing to the similarity of these substances, and as required by REACH, these substances have been grouped into the resin oils and cyclic dienes category containing 15 complex UVCB substances, coded LOA-01 to LOA-12 and BASF LOA-1–BASF LOA-3.

**Table 1:**
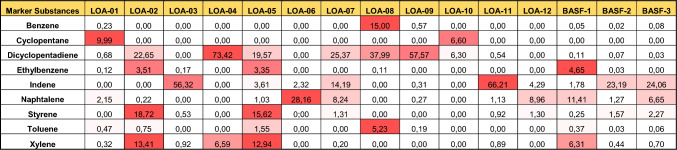
Relative concentrations (in %(w/w)) of major constituents (marker compounds) of the 15 tested resin oils and cyclic dienes UVCB substances

The intensity of the red color denotes the relative composition of the marker substance across streams

Table 1 shows the relative concentrations of the most important, data-rich, individual chemicals, which at least partly make up the composition of these substances. Considering the variability of the substances, the question arises which of the two previously mentioned approaches for chemical grouping under REACH would be more appropriate or if better options could be considered.

To improve the quality of chemical grouping and read-across, the RAAF encourages the submission of supporting evidence. Several initiatives have demonstrated how NAMs could be used for such purposes (Pestana, 2021). However, most of these methods are in vitro and are often problematic for UVCB substances, and they do not consider the current regulatory reality in which in vivo data form the major basis of risk assessment. The quality and reliability of chemical grouping could be improved by making better use of these regulatory-required studies by extracting more data by means of ’omics technologies. Thus, information obtained from classical in vivo data (clinical observations, clinical pathology, and histopathology) and ’omics information can be jointly used to improve the quality of a read-across as suggested by van Ravenzwaay et al. (2012). The introduction of’ omics technologies, where data on large numbers of distinct molecular endpoints are generated simultaneously, has provided several tools that can improve the safety assessment process (Bugrim 2004). By examining the effects of compounds on mRNA (transcriptomics), proteins (proteomics), or endogenous metabolites (metabolomics), subtle changes presaging overt toxicity can be detected. The latter technique has seen application in analyzing biofluids such as urine and blood and, as such, has the capability of querying systemic perturbations in the entire organism after treatment (Robertson 2005). Metabolomics has been used to identify biomarkers for disease state, drug effect, and toxicity (Boudonck 2009). Metabolite profiling can also be used for pattern recognition approaches wherein the responses of several signals are collectively used to characterize a particular state or response (Nicholson 1999). Such pattern recognition approaches are most accurate when the reference patterns are based on a large database of profiles collected under controlled conditions (Strauss et al. 2009).

In a case study using metabolomics data from 28-day studies in rats with phenoxy herbicides, it was shown how such a biological-based approach could be used to choose the best read-across option from various candidates to waive the requirement for a 90-day study (van Ravenzwaay et al. 2016).

The purpose of this study was to investigate the utility of metabolomics for chemical grouping and serve as a 14-day dose range finding study for potential additional regulatory studies with longer duration. To this aim, the 15 UVCBs mentioned in Table 1 were administered orally to rats, and in addition to clinical pathological and histopathological investigations, plasma metabolomics were performed. Combining these data, it was evaluated whether subsets of the most similar of these UVCBs could be identified, based on this biologically based grouping approach. Proof of concept of such a methodology could serve as an example of how ECHA’s substance-based approach, in combination with an in vivo-based NAM (metabolomics), could be used to reduce the amount of animal testing by forming biologically similar subsets of UVCBs and to test only representative substances, rather than all substances in a given category.

## Materials and methods

### Ethics statement

The studies were approved by the BASF Animal Welfare Body and performed according to the German Animal Welfare Act and EU Directive 2010/63, with the permission of the local authority, the Landesuntersuchungsamt Rheinland-Pfalz (permission number 23 177-07/G 17-3-063). The laboratory is Association for Assessment and Accreditation of Laboratory Animal Care International (AAALAC)-certified.

### Retention of records

The study was performed in the spirit of GLP. GLP-relevant records and materials are archived at BASF SE for at least the period specified in the GLP principles. This includes the study plan, any amendments, raw data, test/positive/reference item sample specimens (according to test facility SOPs), and the study report.

### Test substances

The test substances used in this study are referred to as LOA resin oils and cyclic dienes (Category L): LOA-01, LOA-02, LOA-03, LOA-04, LOA-05, LOA-06, LOA-07, LOA-08, LOA-09, LOA-10, LOA-11, and LOA-12, as well as BASF LOA substances 1, 2, and 3 (referred to as BASF LOA-1, BASF LOA-2, and BASF LOA-3). The LOA resin oils and cyclic dienes streams were provided by the members of the Lower Olefins and Aromatics (LOA) REACH Consortium, and the BASF compounds, by BASF SE. In addition to these substances, a total of six “marker” substances, i.e., chemicals that are constituents of the various LOA UVCB substances (see Table 1), were tested. These consisted of indene (Sigma-Aldrich, purity 97.8%), cyclopentane (Honeywell, 98.6%), dicyclopentadiene (DCPD) (Sigma-Aldrich, 99.7%), naphthalene (Sigma-Aldrich, 100%), xylene (Sigma-Aldrich, 99.8%), and benzene (Sigma-Aldrich, > 99.9%). In addition to the abovementioned marker substances investigated in this study, metabolome data on compounds such as styrene (purity 99.89%), toluene (purity > 98.5%), and ethylbenzene (purity > 98.5%) were available in BASF’s MetaMap Tox database (see Table 2). These data were obtained in separate studies from the present one; however, the experimental setup including compound administration, rat strain, and environmental conditions, as well as the metabolome data analysis, was identical in the studies.

**Table 2:** Dose levels in mg/kg bw per day for male and female rats

Test substance	High dose	Low dose
LOA-01	1000	300
LOA-02	750	250
LOA-03	500	150
LOA-04	200	70
LOA-05	750	250
LOA-06	1000	300
LOA-07	600	200
LOA-08	300	100
LOA-09	200	70
LOA-10	1000	300
LOA-11	500	150
LOA-12	1000	300
BASF LOA-1	1000	300
BASF LOA-2	1000	300
BASF LOA-3	1000	300
Indene	450	100
Cyclopentane	1000	300
Dicyclopentadiene (DCPD)	150	50
Naphthalene	600	250
Xylene	1000	300
Benzene	1000	300

Based on their chemical composition, the 12 LOA compounds can be subdivided into four major groups:

1. High bicyclic olefin content with low-to-medium aromatic content: LOA-04, LOA-08, LOA-09, and LOA-10
2. Intermediate bicyclic olefin content and moderate high bicyclic olefin content: LOA-01 and LOA-07
3. Moderate high bicyclic olefin content: LOA-02 and LOA-05
4. High aromatic cyclic olefins, diolefins, and alkanes: LOA-03, LOA-06, LOA-11, and LOA-12

Corn oil (obtained from Caelo) was used to prepare the solutions for oral gavage administration.

### Animal maintenance and administration

Wistar rats (Crl:WI(Han)) were obtained from Charles River Laboratories, Sulzfeld, Germany. At the beginning of the treatment, the age of the animals was 60 ± 5 days. The animals were housed together (five animals per cage) in H-Temp polysulfonate cage type 2000P. Dust-free wooden bedding was used, and wooden gnawing blocks (LIGNOCEL^®^ block large) were added for environmental enrichment. The animals were accommodated in air-conditioned rooms with a uniform temperature of 20–24 °C and a relative humidity of 45–65%. The day/night cycle was 12 h. Diet and drinking water were available ad libitum (except before blood sampling) and regularly assayed for chemical contaminants and microorganisms. There were five animals per sex in the treatment group and 10 animals per sex in the control group. The animals were treated with the test compounds orally, by gavage (volume 4 mL/kg bw) daily for 14 days. Test substances were prepared weekly as a solution in corn oil divided in daily portions and stored in the refrigerator until daily use.

### Dose levels

The dose levels were selected based on a 7-day repeated dose range finding experiment, in which either the limit dose (1000 mg/kg bw), or a dose as high as possible, avoiding severe suffering or lethality, was targeted. Further criteria for high dose selection were clinical observations in the first days but not at the end of administration period such as (semi)-closed eyelid and apathy. Based on the results obtained, the following doses were selected for both males and females.

### Dose route

The oral route was selected as this is the preferred route by ECHA under REACH and has been used to establish the toxicological profile of the test substances, marker compounds, and substances in the metabolome database MetaMap Tox.

### Animal examinations and sampling

Although no regulatory test guideline exists for this type of study, for parts of the study, i.e., clinical examinations, clinical pathology, and histopathology, reference is made to the OECD Guideline for Testing of Chemicals, Method No. 407: Repeated Dose 28-Day Oral Toxicity Study in Rodents. A detailed list of all examinations is provided in supplementary data # 1. Briefly, the following parameters were determined: mortality, clinical signs of toxicity, body weight, food consumption, hematology, clinical chemistry, organ weights, macroscopic pathology, and histopathology. On the morning of the sacrifice, blood was taken from the retrobulbar venous plexus of fasted animals. The animals were anesthetized using isoflurane. The blood sampling procedure and subsequent analysis of blood and serum samples were carried out in a randomized sequence.

### Metabolome analysis

Plasma metabolome analyses were performed by BASF metabolome solutions GmbH (Berlin, Germany) using GC–MS and LC–MS/MS techniques. From 60 μL rat plasma, metabolites were extracted with a mixture of methanol, dichloromethane, water, and toluene (93:47:16.5:1, v/v) buffered with ammonium acetate. Internal standards were added to the extraction mixture to ensure reproducible analysis. After centrifugation, an aliquot of the extract was subjected to LC–MS/MS analysis using reverse phase and hydrophilic interaction liquid chromatography (HILIC) followed by MS/MS detection (AB Sciex QTRAP 6500 +) using the positive and negative ionization modes. For RP-HPLC, gradient elution was performed with water/methanol/0.1 M ammonium formate (1:1:0.02 w/w) and methyl-tert butyl ether/2-propanol/methanol/0.1 M ammonium formate (2:1:0.5:0.035 w/w) with 0.5 wt% formic acid. HILIC gradient elution was performed with acetonitrile with 1 vol% water and 0.2 vol% acetic acid (A) and 0.007 M ammonium acetate with 0.2 vol% acetic acid (B). A second aliquot of the extract was mixed with water (3.75:1, v/v) resulting in a phase separation. Both phases were analyzed with GC–MS after derivatization. The nonpolar fraction was treated with methanol under acidic conditions to give fatty acid methyl esters that were derived from both free fatty acids and hydrolyzed complex lipids. The polar and nonpolar fractions were further derivatized with O-methyl-hydroxylamine hydrochloride to convert oxo-groups to O-methyl oximes and subsequently with a silylating agent (N-methyl-N-(trimethylsilyl)-trifluoroacetamide). Steroid hormones, catecholamines, and their metabolites were measured by online solid-phase extraction–LC–MS/MS (SPE–LC–MS/MS) (Yamada 2002). Absolute quantification was achieved by means of stable isotope-labeled standards. All samples were analyzed in a randomized analytical sequence to avoid analytical bias. The data were corrected to internal standards and normalized to the median of reference samples, which were derived from a pool generated from sample aliquots of control animals to account for inter- and intra-instrumental variation. For all metabolites, changes were calculated as the ratio of the mean of metabolite levels in individual rats in a treatment group relative to the mean of metabolite levels in rats in a matched control group (time point, dose level, and sex).

### Metabolome evaluation using MetaMap tox

The heteroscedastic t-test (“Welch test”) was applied to determine the statistical significance of metabolite levels between samples from treated animals and respective controls at *p* < 0.05. The percentage of significantly changed metabolites was calculated by dividing the number of significantly changed metabolites by the total number of measured metabolites. Test substance-related changes in the metabolome were analyzed as follows:

1. Analysis of specific metabolic changes for each dose group.
2. A similarity analysis of the test compound’s metabolic profile with predefined patterns in MetaMap^®^ Tox (> 110 patterns currently covering 42 modes of action and more than 70 different toxicological effects) was determined with an algorithm using a median r-value metric. Subsequently, these were evaluated by a BASF internal expert panel. A (good) match prerequisites 90% or more of metabolites significantly changed in the same direction as defined by the pattern (weak match: 75– < 90%; equivocal result: 50– < 75%; and mismatch: < 50%). An example of such a process for phenytoin can be found in Kamp et al. (2012).
3. A comparison of the entire metabolomic profiles of the test substance with those of all the compounds available in MetaMap Tox (ca. 1000) using Spearman and Pearson correlations. This is obtained by calculating all pairwise coefficients of the entire database stratified by sex (male/female) and dose (high/low). A threshold value of 0.40 for male animals and 0.50 for female animals is equivalent to the 95th percentile of all correlation coefficients. Correlation coefficients at or above these values are considered biologically relevant.

In addition to the abovementioned standard procedure for metabolome evaluation, for the purpose of this study, i.e., to evaluate whether grouping of the test substances based on their metabolome would be possible, several additional analyses were performed.

1. Treatment correlation-based clustering: Pearson’s correlation values between all high-dose treatment pairs were obtained from MetaMap Tox (for details, see point 3 above) and transformed into a vector per treatment stratified by sex. Using these vectors, a hierarchical clustering analysis using the Euclidean distance metric and ward.D2 clustering method was performed. For visualization, heatmaps per sex were plotted and the treatments were ordered according to the clustering results.
2. Hierarchical clustering analysis using full metabolic profiles was performed using the Manhattan distance metric and ward.D2 clustering method. For this, the median value (log-transformed data) for each metabolite per treatment group was computed from individual animal data. The data were further centered relative to controls, by subtracting the median value of all controls per analyte.
3. Principal component analysis (PCA) was performed on log-transformed single animal data by centering (with respect to controls, obtained by subtracting the median value of all control samples per metabolite from the individual animal values) and scaling to unit variance. To generate artificial samples, a bootstrap sampling algorithm was used. A pseudo-animal metabolic profile was generated by independent sampling, with replacement, individual metabolite data restricted within a treatment group, stratified by sex, dose, and measurement day. The resulting metabolic profile contains, for each metabolite, a value that is randomly selected from one of the animals of the respective treatment group. This procedure was repeated 100 times for each group to get 100 pseudo-animals. The sample procedure reduces the impact of outliers. As each metabolite is independently sampled, it breaks any inter-metabolite correlations, but as the sampling is restricted within a group it preserved intergroup variabilities. PCAs using original and bootstrap data were performed.

### Statistics

For clinical examinations, the two-sided Dunnett’s test was used. For all organ weight parameters and those hematology and clinical pathology parameters with bidirectional changes, the nonparametric one-way Kruskal–Wallis test was applied. If the resulting *p*-value was ≤ 0.05, a pairwise comparison of each dose group with the control group was performed using the Wilcoxon test (two-sided). For parameters with unidirectional changes, the Wilcoxon test (one-sided) was used.

## Results

### Toxicological parameters

The results of the toxicological investigations (clinical observations, clinical pathology, and histopathology) can be found for the UVCB substances, and the marker compounds are provided in supplementary material # 2a and 2b. A brief overview of changes for the high dose of all tested substances is shown in Table 3.

**Table 3:**
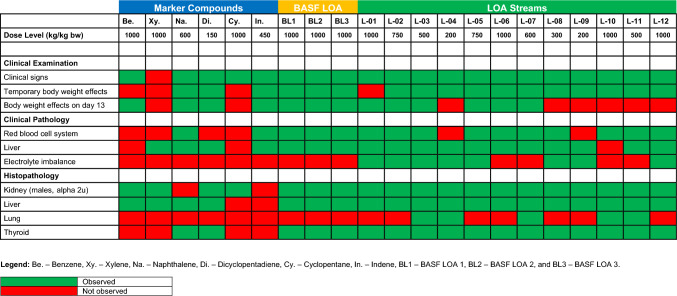
Results of clinical examinations, clinical pathology, and histopathology

Overall, these investigations demonstrated a remarkable similarity of findings in the 12 LOA substances and the three BASF substances. At the high dose level, for both sexes, signs of clinical toxicity were noted for all compounds. The dose levels at which toxicity was observed were the lowest for DCPD and the two DCPD-rich substances (LOA-04 and LOA-09). These consisted mainly of transient (usually limited to the first 3 days of administration) symptoms such as apatheia, (semi) closed eyelids, piloerection, and occasionally lacrimation. Reduced food consumption and reduced body weight gain were noted for all substances. In contrast, a reduction in body weight at the end of the administration period was only noted in males, for 10 of the 15 substances. Clinical chemistry often demonstrated increased cholesterol, triglycerides, and alanine aminotransferase activity (ALT) and occasionally increased γ-glutamyl transferase activity (GGT) effects associated with changes in liver function. Nearly all compounds induced changes in hematology, indicative of effects on the red blood cell system. The most frequently changed parameters were increased reticulocyte counts, occasionally associated with decreased hemoglobin, hematocrit, and mean corpuscular hemoglobin concentration (MCHC), and changes in prothrombin time. Overall, this can be interpreted as increased red blood cell turnover and regenerative anemia.

At the high-dose level, increased organ weights were mainly noted in the liver, thyroid, and kidneys (males only). Histopathological investigations demonstrated some liver changes for all 15 substances. Most commonly, they were diagnosed as diffuse or centrilobular liver cell hypertrophy, with gradings between minimal and severe. Occasionally, these changes were accompanied by prominent nucleoli. For the three BASF substances, liver pathology changes were only observed in female rats, whereas for the LOA substances they were seen in both males and females. The changes were interpreted as the result of liver enzyme induction. Histopathological effects observed in the thyroid were noted in seven of the 12 LOA substances and in all BASF substances, mostly in both sexes, and consisted of follicular cell hypertrophy. In conjunction with the finding in the liver, thyroid pathology was assessed to be associated with a mechanism consistent with the increased metabolism and excretion of thyroid hormones based on increased liver enzyme activity and a subsequent increase in thyroid-stimulating hormone, which induces the thyroid to produce greater levels of thyroid hormone. This effect is reversible, and rats are known to be particularly sensitive (Vansell 2022). The kidneys of male rats also show toxicity caused by a rat-specific production of a protein called alpha 2u-globulin, which accumulates in the rat kidney and causes nephron toxicity (Swenberg 1993).

The toxicity observed with the six marker substances at the high-dose level was overall rather similar to that of the 15 UVCB substances; however, with a tendency to be less pronounced, e.g., cyclopentane was virtually without any relevant toxicity. There were no effects on electrolyte levels and hematological changes observed for indene and naphthalene. The most common findings among the marker compounds were changes in the liver, substantiated by increased weights and pathology, which again was generally like the UVCB substances.

### Metabolomics

The results of the metabolome analysis are presented with the focus on providing useful information for chemical grouping, i.e., to evaluate the extent of similarity, or absence thereof, between the profiles of the 15 UVCB substances and the marker substances. Generally, strong, dose-dependent effects on the plasma metabolome were seen for all compounds in male and female animals. Only minimal effects were observed for cyclopentane even at high dose (both sexes).

### Univariate analysis—pattern ranking

At the time of the evaluation of the metabolome results, the BASF metabolomics database contained more than 110 predefined patterns, representing 42 specific modes of action or forms of organ toxicity. The metabolome profiles of all LOA and BASF compounds were compared against all these patterns. The individual metabolome changes in these substances can be found in supplementary # 3 material.

Overall, matches and weak matches (i.e., a concordance of metabolome changes in the profile of the test substances with > 75% of those metabolites in the predefined patterns) were noted for one or more substances for modes of action associated with liver, kidneys, thyroid, and hematological parameters. In male and female livers, the following associations were noted for at least one of the substances: toxicity, paracetamol-like toxicity, enzyme induction, and short-chain phthalate-like toxicity. For females, in addition, a putative pattern for cholestasis was indicated. In the male and female kidneys, a pattern consistent with interstitial nephritis was observed. Additionally, for females, two more patterns associated with kidney function were noted: tubular toxicity and inhibition of the organic anion transporter. For the thyroid, an indirect effect related to increased excretion of thyroid hormones due to liver enzyme induction and increased thyroid hormone excretion, was indicated for both sexes. The ability of metabolomics, in combination with the MetaMap Tox database to identify such modes of action on the thyroid, was shown by Montoya et al. (2014). For hematological parameters, a pattern of anemia and bone marrow suppression was observed in males and platelet aggregation inhibition in females. Based on the good concordance with the predefined patterns, it is reasonable to assume that the abovementioned patterns have a high likelihood of being predictive of the respective mode of action. One pattern that was observed for one compound in males called “serotonin reuptake inhibition,” a pharmacological mode of action, is difficult to interpret but may have been associated with the concomitant liver enzyme-inducing properties of the reference compounds. Additionally, this pattern did not correlate with any classical parameters in the study and was therefore considered to be incidental.

From a regulatory point of view, confidence in the absence of toxicity for substances under evaluation for chemical grouping is just as important as the presence of toxicological effects. The pattern ranking indicated that not more than 12 patterns with matches or weak matches were positively linked to the administration of the 15 UVCB substances. As there are more than 110 patterns in the MetaMap Tox database to which a link could have been established, this means that there were no matches for any of these substances with nearly 100 other patterns (for details, see supplementary material # 4). Importantly, there were no additional metabolome patterns identified for the UVCB substances and they were also not seen with the marker substances.

### Univariate analysis—profile comparison and clustering

Profile comparisons between all high-dose treatment pairs were obtained from MetaMap^®^Tox, which contains > 1000 compounds. The profile comparison showed that there are strong similarities within the LOA and BASF substances. Correlations with compounds in MetaMap^®^ Tox, above the threshold levels of relevance, i.e., a Pearson correlation coefficient above 0.4 for males and above 0.5 for females, representing the 95th percentile of all possible correlations in the database, were observed with compounds that have predominantly the liver as the target organ.

**Fig. 1:**
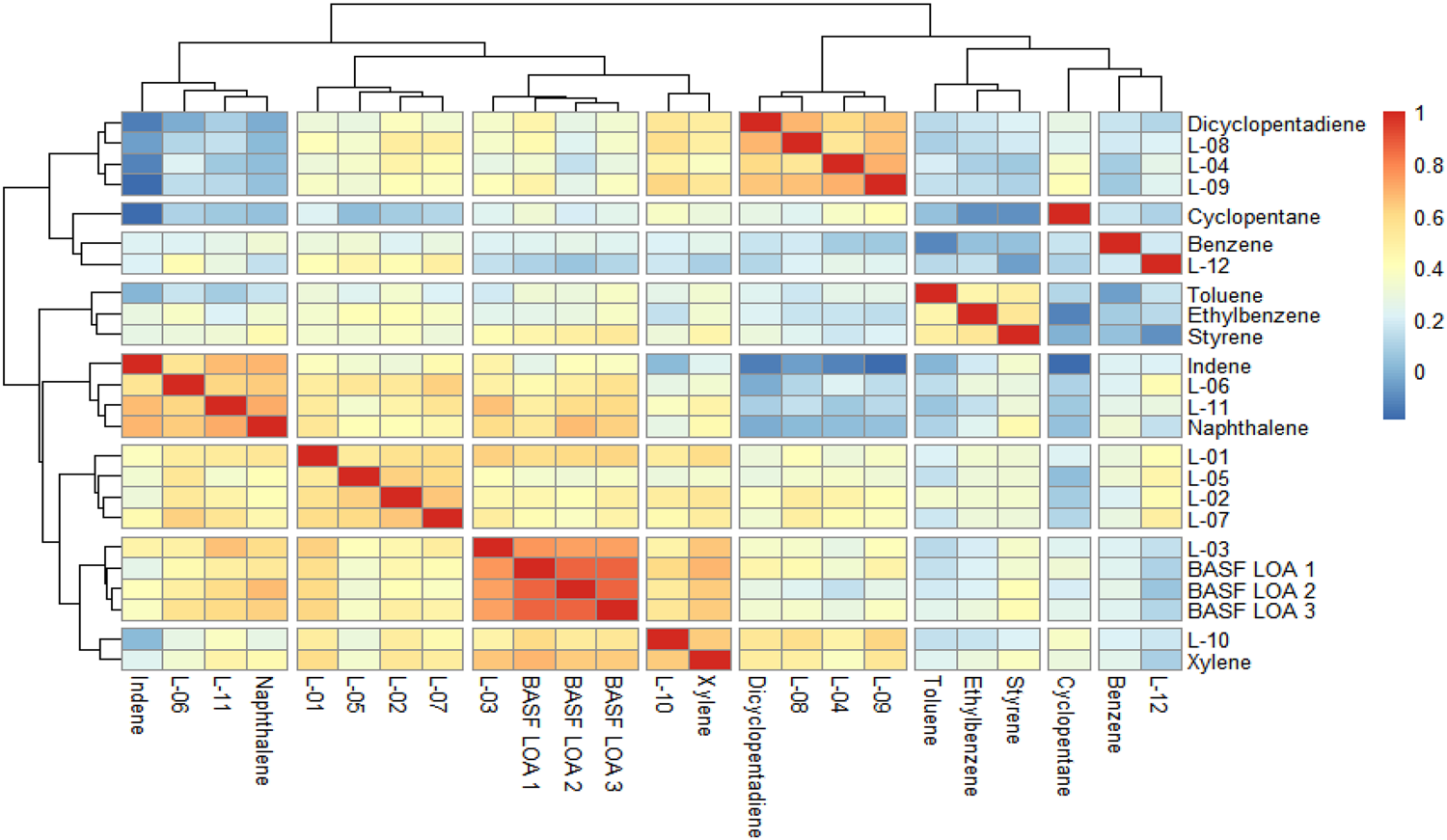
Univariate clustering analysis based on correlation coefficients for males *(L-01–12 in the figure represents LOA-01–12)*. Color coding indicates the strength of the correlation from negative (dark blue) to 1 (red)

**Fig. 2:**
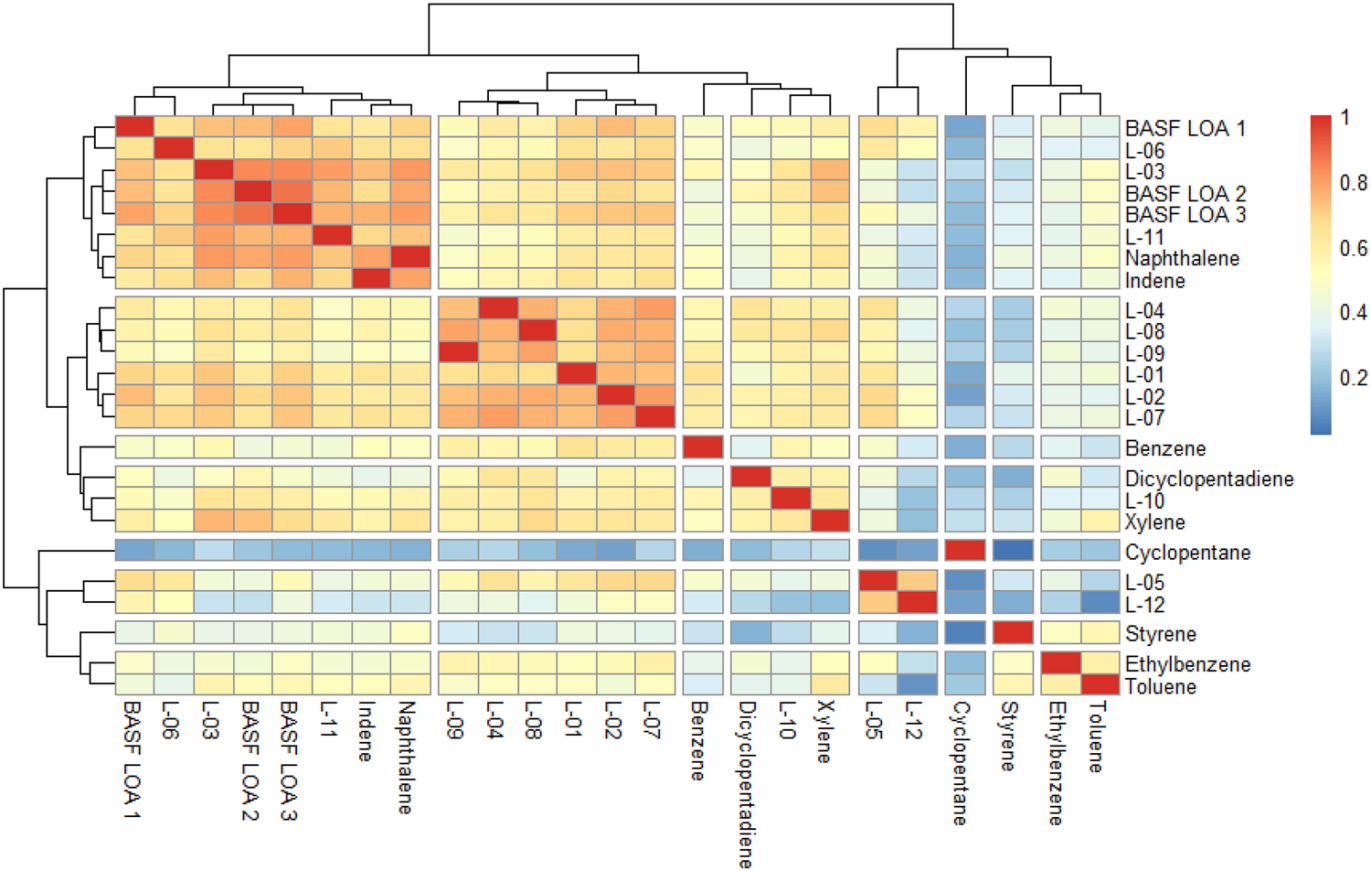
Univariate clustering analysis based on correlation coefficients for females. *(L-01–12 in the figure represents LOA-01–12).* Color coding indicates the strength of the correlation from negative (dark blue) to 1 (red)

The profile comparison between the study compounds revealed some clusters of high similarity within the data set, as expressed in their correlation coefficients (Figs. 1 and 2).

Based on the obtained correlation coefficients, the following clusters of higher similarity within the data set were observed for male animals:

Cluster 1: LOA-04, LOA-08, and LOA-09, which were shared with dicyclopentadiene.

Cluster 2: LOA-06 and LOA-11, which were shared with indene and naphthalene.

Cluster 3: LOA-01, LOA-02, LOA-05, and LOA-07.

Cluster 4: BASF LOA-1, BASF LOA-2, BASF LOA-3, and LOA-03.

LOA-10 clustered with xylene.

For females, the correlation coefficient produced the following clusters:

Cluster 1: LOA-01, LOA-02, LOA-04, LOA-07, LOA-08, and LOA-09.

Cluster 2: LOA-05 and LOA-12.

Cluster 3: BASF LOA-1, BASF LOA-2, and BASF LOA-3 and LOA-03, LOA-06, and LOA-11.

Cluster 4: LOA-10, DCPD, and xylene.

### Multivariate analysis—hierarchical clustering

**Fig. 3:**
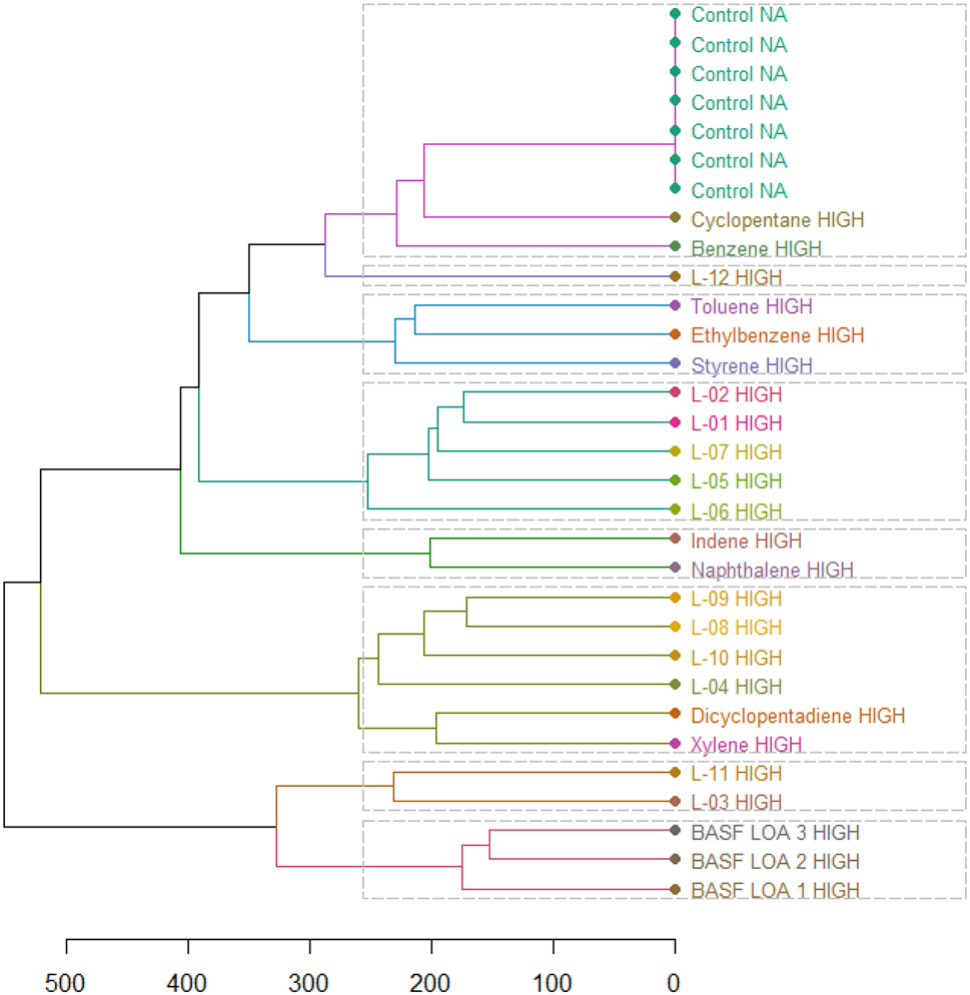
Multivariate hierarchical clustering analysis for males. *(L-01–12 in the figure represents LOA-01–12)*

**Fig. 4:**
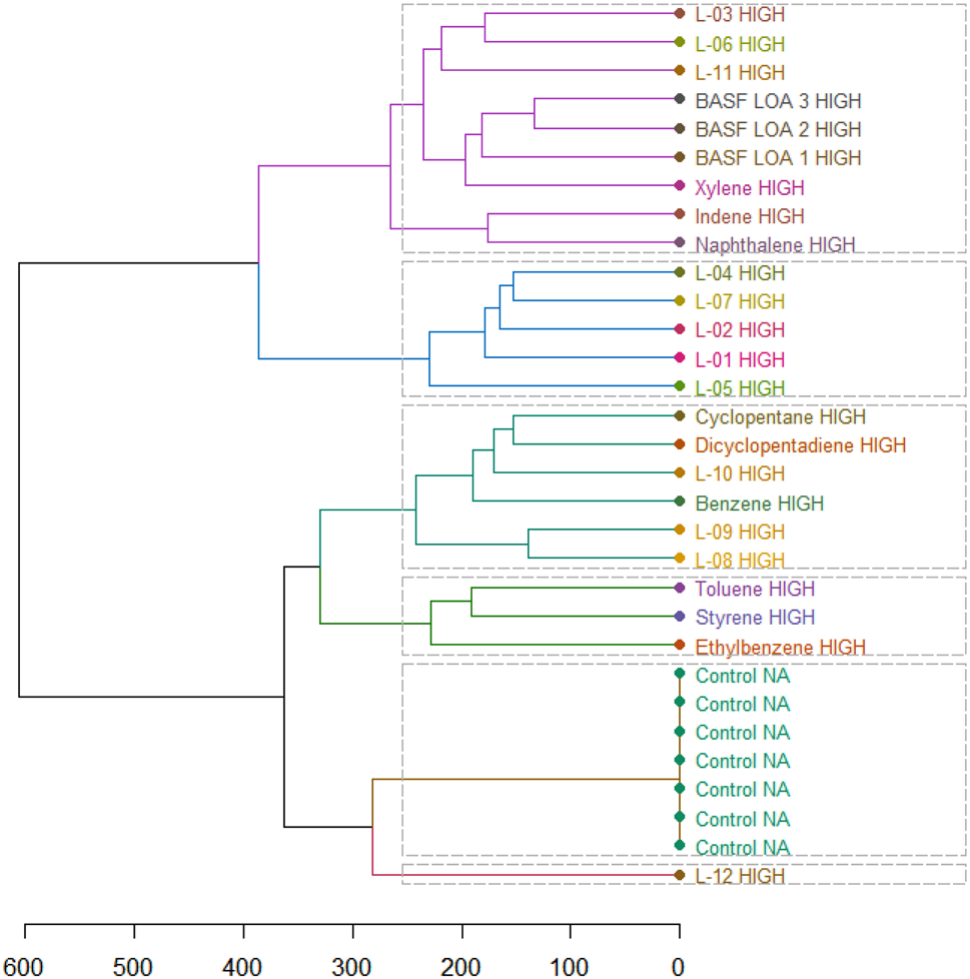
Multivariate hierarchical clustering analysis for females. *(L-01–12 in the figure represents LOA-01–12*)

To further investigate potential (sub)clusters within the test substance high dose,

hierarchical clustering was performed. The results thereof can be seen in Figs. 3 and 4

The hierarchical clustering for male animals showed the following (sub)clusters.

Cluster 1: LOA-04, LOA-08, LOA-09, and LOA-10 and closely related dicyclopentadiene and xylene.

Cluster 2: Indene and naphthalene.

Cluster 3: LOA-01, LOA-02, LOA-05, LOA-06, and LOA-07.

Cluster 4: BASF LOA-1, BASF LOA-2, BASF LOA-3, and closely related LOA-03 and LOA-11.

For LOA-12, there was no apparent clustering.

The hierarchical clustering for high-dose females showed the following (sub)clusters.

Cluster 1: LOA-1, LOA-2, LOA-04, LOA-05, and LOA-07.

Cluster 2: The subcluster formed by LOA-08 and LOA-09 was closely related to a subcluster formed by LOA-10 and cyclopentane, dicyclopentadiene, and benzene.

Cluster 3: The subcluster formed by BASF LOA-1, BASF LOA-2, and BASF LOA-3 was closely related to the subcluster consisting of LOA-03, LOA-06, and LOA-11. These subclusters were most similar to xylene, indene, and naphthalene.

For LOA-12, there was no apparent clustering; however, this compound was close to controls.

### Multivariate analysis—principal component analysis (PCA)

**Fig. 5:**
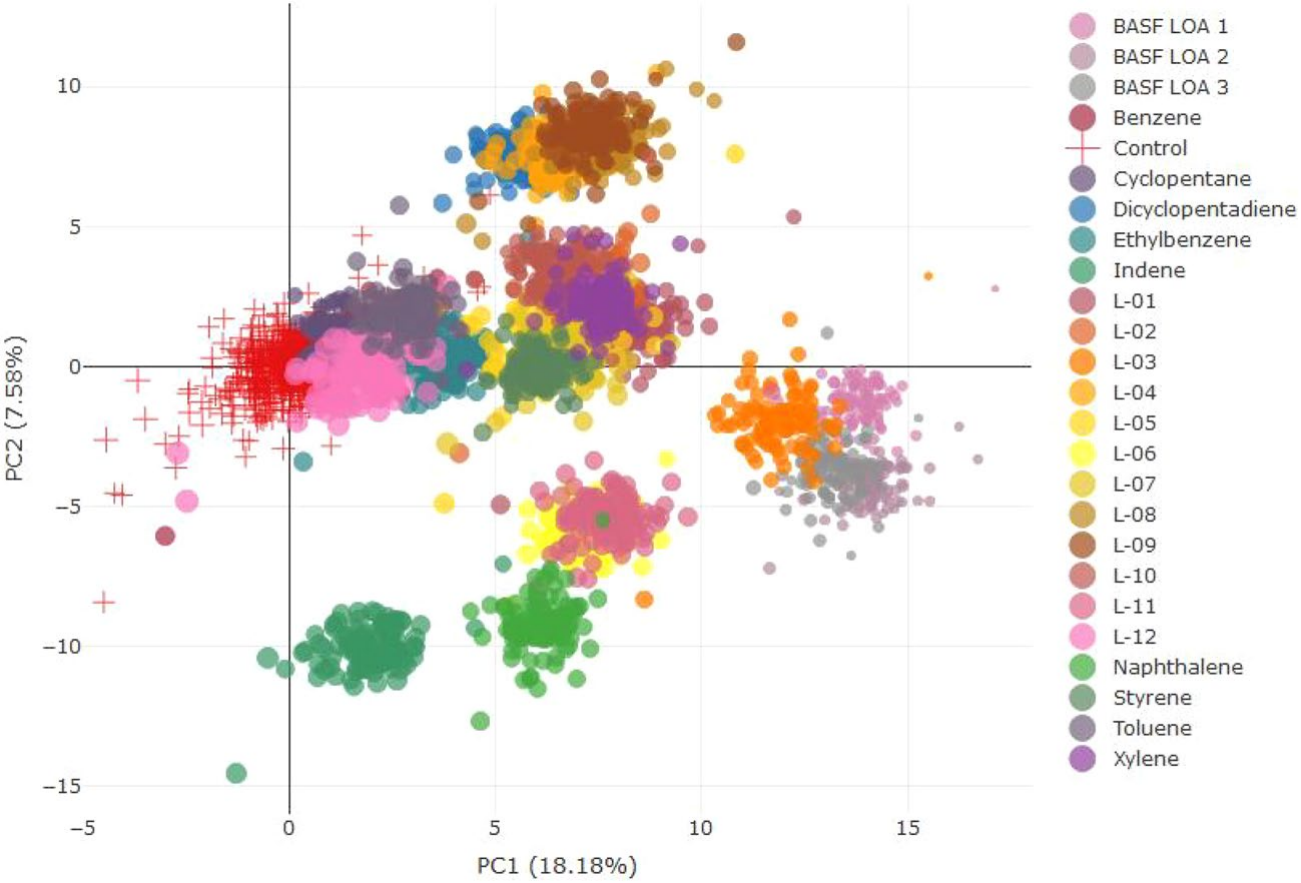
Bootstrapped PCA of all compounds and marker substances for males. *(L-01–12 in the figure represents LOA-01–12)*

**Fig. 6:**
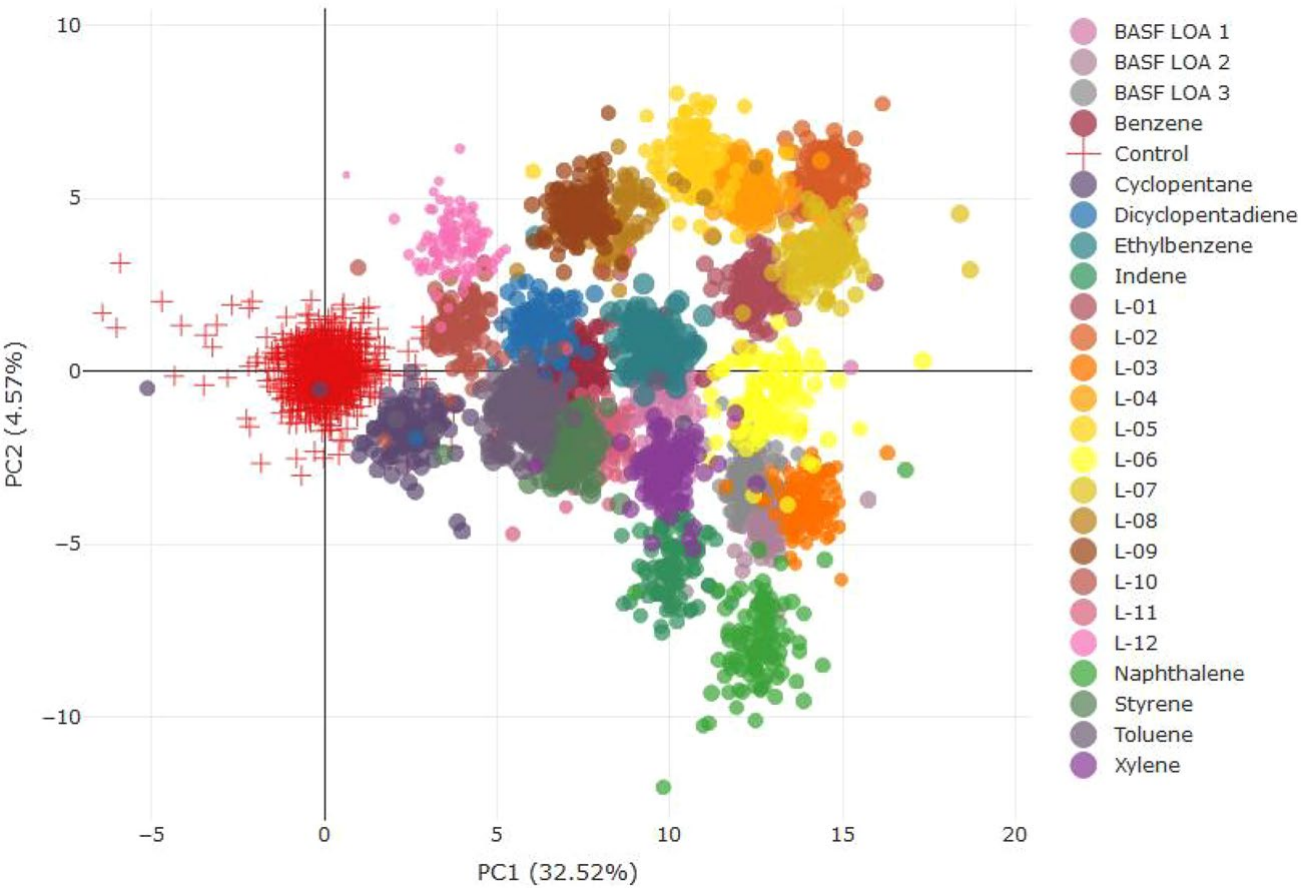
Bootstrapped PCA of all compounds and marker substances for females. *(L-01–12 in the figure represents LOA-01–12*)

A separate way to investigate the clustering of substances is by means of PCA. This type of analysis allows the visualization of high-dimensional data in few dimensions. Through the linear transformation of the original variables by making a rotation of the multidimensional space, as much variation as possible is retained along the so-called principal components (PCs). Consequently, the first PC carries the most information. With this technique, differences can be identified, and samples can be grouped based on the similarity of differences. Considering that the number of samples was rather low for such a representation (each compound would be represented by five individual points per sex and dose), a bootstrap procedure was used. This procedure uses the individual data and their variability within each treatment group and controls, to generate a virtual data set, containing a high number of bootstrapped samples (100 pseudo-animals per group were generated, additionally the original animals) to visualize the space within the PCA occupied by the respective treatment groups. These data are shown in Figs. 5 and 6.

The reason for the reduction in explained variation is that the bootstrap sampling procedure adopted here breaks correlations between metabolites (within a treatment group, while conserving intra-group) resulting in an increased randomness in the data. This results in a decrease in the variation explained by the first few PCs. This visualization allows for the identification of four clearly separated clusters:

Cluster 1: LOA-04, LOA-08, and LOA-09 clustering with the marker compound DCPD.

Cluster 2: LOA-06 and LOA-11 clustering with naphthalene and indene.

Cluster 3: LOA-01, LOA-02, LOA-05, LOA-07, and LOA-10.

Cluster 4: BASF LOA-01, BASF LOA-02, BASF LOA-03, and LOA-03.

LOA-12, Benzene, cyclopentane, and ethylbenzene also appear as a cluster; however, they are positioned close to the controls.

The PCA visualization for females appears to be more compound-specific and less clustered than for males. Two clusters may be identified:

Cluster 1: LOA-01, LOA-02, LOA-04, LOA-05, LOA-07, LOA-08, and LOA-09.

Cluster 2: LOA-10, LOA-12, and cyclopentane.

Cluster 3: BASF LOA-01, BASF LOA-02, BASF LOA-03, LOA-03, LOA-06, and LOA-11, which clustered with the marker substances such as naphthalene, indene, and xylene.

LOA-10, LOA-12, and cyclopentane were close to the controls.

## Discussion

The purpose of the present study was to ascertain whether a plasma metabolome analysis performed within the context of regulatory toxicity testing could aid in the grouping of chemicals for UVCB substances. In addition, based on the combined evaluation of all the data, a proposal for grouping the biologically most similar subsets of UVCBs is made. Two aspects need to be considered. As evidenced by the results of this study, all tested UVCB substances have rather similar forms of toxicity and therefore can be seen as one category and further grouping will be based on subtle subclusters rather than on profoundly different types of toxicity. Moreover, as the metabolome of rats and their metabolomic response to toxicity are known to be sex-specific, the consideration of the results will be done for males (m) and females (f) separately (Strauss et al. 2009).

### Classical toxicity parameters

The LOA streams showed qualitatively a relative homogenous toxicity with liver, thyroid, and hematopoietic system as primary target organs in both sexes. The observed effects are indicative of hepatic enzyme induction, secondarily leading to increased excretion of thyroid hormones, an effect that is likely not relevant for humans (Hall et al. 2012; McClain 1989). In male animals, alpha 2u-globulin accumulation was observed in the kidney, an effect that is also not human-relevant (Swenberg, 1993a). The toxicity induced by the marker compounds is less homogenous; however, when compared to the LOA streams, no additional toxicities were observed. It is concluded that the toxicological parameters do not provide a sound basis for clustering within this rather similar group of UVCB substances.

### Metabolomics

The use of metabolomics has matured over the last years, and best practices, including its use for chemical grouping and reporting standards, are now available (Harrill et al. 2021; Viant et al. 2019). Before addressing the metabolome observations in detail, the results thereof indicate that for both univariate and multivariate analyses, clusters were obtained and that consequently this information is more suitable for biology-based grouping purposes than classical toxicology alone. Considering the sex-specific nature of the metabolome, results and conclusions will be discussed for males and females separately.

All types of statistical analysis provided indications of the clustering of the substances, frequently in association with one or more of the marker compounds. Whereas the univariate and multivariate hierarchical clustering analyses provide information on similarities, the multivariate PCA focuses on differences between the treatment groups and thus provides a different perspective. Tables 4 and 5 provide an overview of the clusters for each of the statistical methods for males and females, respectively.

**Table 4:** Summary of clustering results based on univariate and multivariate statistical methods for males

Univariate—coefficient correlation			
Cluster 1	Cluster 2	Cluster 3	Cluster 4
LOA-04	LOA-06	LOA-01	LOA-03
LOA-08	LOA-11	LOA-02	BASF LOA-1
LOA-09	Indene	LOA-05	BASF LOA-2
DCPD	Naphthalene	LOA-07	BASF LOA-3
Multivariate—hierarchical clustering			
Cluster 1	Cluster 2	Cluster 3	Cluster 4
LOA-04	Indene	LOA-01	BASF LOA-1
LOA-08	Naphthalene	LOA-02	BASF LOA-2
LOA-09		LOA-05	BASF LOA-3
LOA-10		LOA-06	Subcluster
Subcluster		LOA-07	LOA-03
DCPD			LOA-11
Xylene			
Multivariate—principal component analysis			
Cluster 1	Cluster 2	Cluster 3	Cluster 4
LOA-04	LOA-06	LOA-01	LOA-03
LOA-08	LOA-11	LOA-02	BASF LOA-1
LOA-09	Indene	LOA-05	BASF LOA-2
DCPD	Naphthalene	LOA-07	BASF LOA-3
		LOA-10

**Table 5:** Summary of clustering results based on univariate and multivariate statistical methods for females

Univariate—coefficient correlation			
Cluster 1	Cluster 2	Cluster 3	Cluster 4
LOA-01	LOA-05	BASF LOA-1	LOA-10
LOA-02	LOA-12	BASF LOA-2	DCPD
LOA-04		BASF LOA-3	Xylene
LOA-07		LOA-03	
LOA-08		LOA-06	
LOA-09		LOA-11	
Multivariate—hierarchical clustering			
Cluster 1	Cluster 2	Cluster 3	
LOA-01	LOA-08	BASF LOA-1	
LOA-02	LOA-09	BASF LOA-2	
LOA-04	Subcluster	BASF LOA-3	
LOA-05	LOA-10	Subcluster	
LOA-07	Cyclopentane	LOA-03	
	DCPD	LOA-06	
	Benzene	LOA-11	
		Xylene	
		Indene	
		Naphthalene	
Multivariate—principal component analysis			
Cluster 1	Cluster 2	Cluster 3	
LOA-01	LOA-10	BASF LOA-1	
LOA-02	LOA-12	BASF LOA-2	
LOA-04	Cyclopentane	BASF LOA-3	
LOA-05		LOA-03	
LOA-07		LOA-06	
LOA-08		LOA-11	
LOA-09		Xylene	
		Indene	
		Naphthalene	

The clustering results for both sexes are similar, but not identical, which is in line with previous reports indicating an influence of sex on the metabolome profile of rats (Strauss et al. 2009). Comparing the clustering of the substances for the univariate and multivariate analyses, there is a reasonable consistency, with a few differences. Those few that are identifiable, e.g., LOA-04 and LOA-09 in females having no liver pathological effects, and the three BASF LOA substances that have no effects on electrolytes, are identified as clusters with the statistical approaches.

It was also evaluated whether there is clustering with the marker compounds, i.e., if those substances that contain the highest amount of a particular marker compound cluster together with that marker compound. The concentrations of the marker compounds, shown in Table 1, indicate that only for DCPD and indene the concentrations in the UVCBs were dominant. Thus, from the chemical perspective the following clustering could be predicted (marker compound content in parenthesis):

1. A DCPD group consisting of LOA-04 (73%) and LOA-09 (58%), potentially clustering with LOA-08 (38%) and LOA-07 (25%)
2. An indene group consisting of LOA-03 (56%) and LOA-11 (66%) potentially clustering with BASF LOA-2 (23%) and BASF LOA-3 (24%)

Comparing these predictions with the biological analysis, chemical composition contributes to clustering but is not the only decisive factor. For males, the statistical analyses identified a DCPD group, which included LOA-04, LOA-08, and LOA-09. For females, a clear DCPD cluster was not evident; only the hierarchical approach clustered DCPD with LOA-08 and LOA-09.

For males, indene clustered with LOA-11, but not with LOA-03. For females, the indene group was more visible and hierarchical, and PCA clustering grouped LOA-03 and LOA-11 and BASF LOA-2 and BASF LOA-3 compounds in an indene group.

Our investigations demonstrate that using metabolome data, in contrast to toxicological parameters, a grouping approach to find the most similar substances in the category is possible. As phenotypic anchoring in this study is not possible, the reliability of the derived clusters needs to be discussed. The observation that there are two clusters, which correspond to concentration-wise dominant marker substances, is an indication that the metabolome grouping approach provides reasonable answers. The use of metabolomics was successful in identifying, within a group of structurally and toxicologically comparable phenoxy herbicides, the most similar one (van Ravenzwaay et al. 2016). Another example of a metabolomics-based grouping approach was published for amino ethanol (Sperber et al. 2019). An overview of such regulatory approaches for metabolomics is provided by Olesti et al., (2021). A further indication of the correctness of our approach is found in the results of the BASF LOA-02 and BASF LOA-03 substances. In all analyses, these two substances cluster most closely together. Chemically, BASF LOA-02 and BASF LOA-03 are within the group of 15 UVCB substances, the ones that are most similar. In fact, they are virtually identical (sharing the same CAS number: 68477–54-3); however, they are produced in two steam crackers at distinct locations. The composition is described as petroleum, steam-cracked C8-12 fraction. The substance chemically most similar to BASF LOA-02 and BASF LOA-03 is BASF LOA-01 described as petroleum, steam-cracked, C9-10 fraction. Indeed, all statistical analysis of the metabolome data places BASF LOA-01 in the same group as BASF LOA-02 and BASF LOA-03. This outcome increases the level of confidence in the grouping approach based on metabolome data.

Although it is not the purpose of this study to recommend chemical grouping for regulatory purposes, a conservative but transparent approach to reconcile the slightly diverging results of the statistical approaches would be to follow the principle of the lowest common denominator of the obtained results. Considering the data in this manuscript, the following groups of substances would be obtained for males and females, respectively.

Males.

Cluster 1: LOA-04, LOA-08, and LOA-09.

Cluster 2: LOA-03, BASF LOA-01, BASF LOA-02, and BASF LOA-03.

Cluster 3: LOA-01, LOA-02, LOA-05, and LOA-07.

Females.

Cluster 1: LOA-03, LOA-06, LOA-11, BASF LOA-01, BASF LOA-02, and BASF LOA-03.

Cluster 2: LOA-01, LOA-02, LOA-4, and LOA-7.

For males, this grouping proposal corresponds reasonably well with the major components of their composition. Cluster 1 contains three of four compounds of the class “high bicyclic olefin content with low-to-medium aromatic content” and could also be described as the high DCPD group. Cluster 2 contains all the indene-rich substances, and cluster 3 has two of the moderate-to-high bicyclic olefin chemicals. For females, in cluster 1 the indene-rich compounds are found together.

In conclusion, a metabolomics approach for chemical grouping is possible and enables to identify groups of most similar substances in a better way than with classical toxicological parameters alone. The use of metabolome database MetaMap^®^ Tox is helpful in identifying organ toxicity and equally important in determining which types of toxicity are not indicated, providing more confidence in the outcome of the correctness of the clusters. However, the database per se is not necessary, as the statistical analysis alone provides an objective way to identify such clusters. Further work would be needed, however, to determine which statistical approaches alone or in combination yield sufficient certainty to justify a read-across. Chemical composition plays a role in clustering, but only when the marker substance is clearly dominant and of significant toxicity, i.e., DCPD and indene. The inclusion of blood-based metabolomics in dose range finding and regulatory studies provides important additional data, which can be used for chemical grouping and could be used to reduce the amount of animal testing on only representative compounds, rather than all. Such additional metabolomics data may also be used for the prediction of toxicological effects. This can help toxicologist and regulators focus on those aspects of the toxicological profile of a compound and may reduce the level of necessary testing to regulate chemicals. When it comes to the effects seen in this study with the 15 tested UVCB substances, beyond signs of clinical toxicity, reduced body weight development, and food consumption, pathological investigation demonstrated the liver, thyroid, kidneys (males only), and hematological system to be the target organs. For the UVCB substances, there were also no metabolome patterns identified that were not seen with the marker substances itself.

### Supplementary Information

Below is the link to the electronic supplementary material.

Supplementary file1 (DOCX 110 KB)

Supplementary file2 (DOCX 32 KB)

Supplementary file3 (DOCX 21 KB)

Supplementary file4 (XLSX 107 KB)

Supplementary file5 (PPTX 125 KB)
