# Supplementary material for: Utility of in vivo metabolomics to support read-across for UVCB substances under REACH
Source: Arch Toxicol. 2024 Jan 24;98(3):755–68. doi: 10.1007/s00204-023-03638-6 (PMC10861390; doi:10.1007/s00204-023-03638-6)
Supplement: Supplementary file 1 — Supplementary file1 (DOCX 110 KB) [file 204_2023_3638_MOESM1_ESM.docx]

Title Pa

Title Page

.

# Analyses

### Food analyses

The supplier assayed the food used in the study for chemical and microbiological contaminants.

### Drinking water analyses

The drinking water is regularly assayed for chemical contaminants by the municipal authorities of Frankenthal and by the Environmental Analytics Water/Steam Monitoring Department of BASF SE as well as for the presence of microorganisms by a contract laboratory.

### Bedding and enrichment analyses

The bedding and the enrichment are regularly assayed for contaminants (chlorinated hydrocarbons and heavy metals) by the supplier.

## Experimental procedureS

Starting on day of arrival, the animals were accustomed to the environmental conditions of the study. Prior to the first detailed clinical observation, the animals were distributed according to weight among the individual test groups, separated by sex. The weight variation of the animals used did not exceed 20 percent of the mean weight of each sex. The list of randomization instructions was compiled with a computer.

The test substance was administered daily by gavage for 14 days. At the end of the administration period blood sampling was carried out and the animals were sacrificed after a fasting period (withdrawal of food) of at least 16 -20 hours.

## Clinical examinations

### Mortality

A check for moribund and dead animals was made twice daily on working days and once daily on Saturdays, Sundays and public holidays. If animals were in a moribund state, they were sacrificed and necropsied.

### Clinical observations

All animals were checked daily for any clinically abnormal signs before the administration, as well as, within 2 hours and within 2-5 hours after the administration. Abnormalities and changes were documented for each animal.

### Food consumption

Food consumption was determined on study days 3, 7, 10 and 13 and calculated as mean food consumption in grams per animal and day.

### Water consumption

Drinking water consumption was determined daily by visual check within the general observation.

### Body weight data

Body weight was determined before the start of the administration period in order to randomize the animals. During the administration period the body weight was determined on day 0 (start of the administration period), 3, 7, 10 and 13. The difference between the respective weighing days and the difference from start of administration (day 0) to end of administration (day 13) was calculated as body weight change.

### Statistics of clinical examinations

Means and standard deviations of each test group were calculated for several parameters (see tables). Further statistical analyses were performed according to following tables:

| **Parameters** | **Statistical test** | **Markers in the tables** | **References** |
| --- | --- | --- | --- |
| body weight,  body weight change | A comparison of each group with the control group was performed using DUNNETT's test (two-sided) for the hypothesis of equal means | * for p ≤ 0.05  ** for p ≤ 0.01 | DUNNETT, C.W. (1955): A multiple comparison procedure for comparing several treatments with a control. JASA, Vol. 50, 1096-1121  DUNNETT, C.W. (1964). New tables for multiple comparisons with a control. Biometrics, Vol. 20, 482-491 |

## Clinical Pathology

On the morning of sacrifice, blood was taken from the retro-bulbar venous plexus from fasted animals. The animals were anaesthetized using isoflurane. The blood sampling procedure and subsequent analysis of blood and serum samples were carried out in a randomized sequence.

The assays of blood and serum parameters were performed under internal laboratory quality control conditions with reference controls to assure reliable test results.

The results of clinical pathology examinations were expressed in International System (SI) units.

The following examinations were carried out in all animals per test group and sex at the end of the administration period.

### Hematology

The following parameters were determined in blood with EDTA‑K_3_ as anticoagulant using a particle counter (Advia 120 model; Bayer, Fernwald, Germany):

Parameters and methods:

| **Parameter** | **Unit** | **Method** | **References** |
| --- | --- | --- | --- |
| Leukocyte count  (WBC) | giga/L | cytochemistry coupled with flow cytometry |  |
| Erythrocyte count  (RBC) | tera/L | flow cytometric laserlight scattering |  |
| Hemoglobin  (HGB) | mmol/L | cyanmethemoglobin method; according to ICSH |  |
| Hematocrit  (HCT) | L/L | calculation:  MCV x erythrocytes |  |
| Mean corpuscular volume  (MCV) | fL | RBC/PLT method; mean of RBC volume distribution curve (histogram) | Operator’s Guide for Advia 120 System |
| Mean corpuscular hemoglobin  (MCH) | fmol | calculation:  hemoglobin  erythrocytes |  |
| Mean corpuscular hemoglobin concentration  (MCHC) | mmol/L | calculation:  hemoglobin  hematocrit |  |
| Platelet count  (PLT) | giga/L | flow cytometric laserlight scattering |  |
| Differential blood count | % and giga/L | cytochemistry coupled with flow cytometry |  |
| Reticulocytes (RETA) | giga/L | cytochemistry coupled with flow cytometry |  |

Furthermore, blood smears were prepared and stained according to WRIGHT without being evaluated, because of non-ambiguous results of the differential blood cell counts measured by the automated instrument. (reference: Hematology: Principles and Procedures, 6^th^ Edition, Brown AB, Lea & Febiger, Philadelphia, 1993, page 101). Only evaluated blood smears were archived.

Clotting tests were carried out using a ball coagulometer (AMAX destiny plus model; Trinity biotech, Lemgo, Germany).

Parameter and method:

| **Parameter** | **Unit** | **Method** | **References** |
| --- | --- | --- | --- |
| Prothrombin time (Hepato Quick’s test)  (HQT) | seconds | citrated blood with calcium thromboplastin | Fischer, M. and Falkensammer, Ch.,  Klin. Wschr. 86, 577-583 (1974) |

### Clinical chemistry

An automatic analyzer (Cobas c501; Roche, Mannheim, Germany) was used to examine the clinicochemical parameters

Parameters and methods:

| **Enzyme (systematic name and system number)** | **Unit** | **Method, wave-length and measuring temperature (Detection limit)** | **References** |
| --- | --- | --- | --- |
| Alanine aminotransferase  (ALT)  (L-alanine: 2-oxoglutarate aminotransferase;  EC 2.6.1.2.) | µkat/L | kinetic UV test, 340 nm; 37°C,  (0.08 µkat/L) | Recommendations of the German Society for Clinical Chemistry: "Standardization of methods for determining enzyme activities in biological liquids". |
| Aspartate aminotransferase  (AST)  (L-aspartate: 2-oxoglutarate aminotransferase;  EC 2.6.1.1.) | µkat/L | kinetic UV test, 340 nm; 37°C,  (0.08 µkat/L) | J. Clin. Chem. Clin. Biochem. 8, 658-660 (1970);  J. Clin. Chem. Clin. Biochem. 9, 464-465 (1971);  J. Clin. Chem. Clin. Biochem. 10, 182-192 (1972) |
| Alkaline phosphatase  (ALP)  (orthophosphoric acid monoester phosphohydrolase;  EC 3.1.3.1.) | µkat/L | kinetic color test, 415 nm, 37°C,  (0.084 µkat/L) | Roche working instructions |
| γ-Glutamyltransferase  (GGT)  (γ -glutamyl) peptide: aminoacid-γ-glutamyl-transferase;  EC 2.3.2.2.) | nkat/L | kinetic color test, 415 nm, 37°C,  (25 nkat/L) | Szasz, G. et al.,  J. Clin. Chem. Clin. Biochem. 12, 228 (1974)  Roche working instructions |

| **Blood Chemistry Parameter** | **Unit** | **Method**  **(Detection limit)** | **References** |
| --- | --- | --- | --- |
| Sodium  (NA) | mmol/L |  |  |
| Potassium  (K) | mmol/L | ion selective electrodes (ISE),  (Na: 80, K: 1.5, Cl: 60 nmol/L) | Roche - working instructions |
| Chloride  (CL) | mmol/L |  |  |
| Inorganic phosphate  (INP) | mmol/L | molybdate reaction  (0.1 mmol/L) | Henry, R.J. in: "Clinical Chemistry", Harper and Row Publishers, New York (1974); Roche working instructions |
| Calcium  (CA) | mmol/L | o-cresolphthalein complex without deproteinization  (0.2 mmol/L) | Ray Sarkar, B.C. and Chauhan, U.P.S., Anal. Biochem. 20, 155 (1967); Roche working instructions |
| Urea  (UREA) | mmol/L | enzymatic determination with the urease/ glutamate dehydro­genase method  (0.5 mmol/L) | Neumann, U. and Ziegenhorn, J.: XVI, Nordiska kongressen for klinisk kemi och klinisk fysiologi 1977, Oulu, Finland; Roche working instructions |
| Creatinine  (CREA) | µmol/L | enzymatic determination with the creatininase/ creatinase /sarcosinoxidase method  (5 µmol/L) | Guder et al., J.Clin.Chem.Clin.Biochem. 24, 889-902 (1986); Roche working instructions |
| Glucose  (GLUC) | mmol/L | hexokinase/glucose-6-phosphate dehydrogenase method  (0.11 mmol/L) | Schmidt, F.H., Klin. Wschr. 39, 1244-1247 (1961); Roche working instructions |
| Total bilirubin  (TBIL) | µmol/L | DPD method  (0.56 µmol/L) | Wahlefeld, A.W. et al., Scand. J. Clin. Lab. Invest. 29, Suppl. 126 (1972) Abstract 11.12; Roche  working instructions |
| Total protein  (TPROT) | g/L | biuret method  (2 g/L) | Luxton, R.W.;Patel, P.Keir, G.Thompson, E.J., Clin Chem 1989, 35 (8), 1731-1734 |
| Albumin  (ALB) | g/L | bromocresol green method  (3.2 g/L) | Doumas et al., Clin. Chim. Acta 31, 87 (1971); Randox working instructions |
| Globulins  (GLOB) | g/L | difference between total protein and albumin |  |
| Triglycerides  (TRIG) | mmol/L | enzymatic color test with lipase esterase/ glycerokinase/ glycerol-3-phosphate oxidase/4-amino-phenazone  (0.1 mmol/L) | mod. method by Wahlefeld, A.W., in "Methoden der enzymatischen Analyse" [Methods of enzymatic analysis] (Bergmeyer, H.U., ed.) Vol. II, 3rd ed., Verlag Chemie Weinheim, GERMANY, pp. 1878-1882 (1974); Roche working instructions |
| Cholesterol  (CHOL) | mmol/L | enzymatic determination with cholesterol esterase/ cholesterol oxidase/4-amino-phenazone (CHOD-PAP method)  (0.1 mmol/L) | Siedel, J. et al., J. Clin. Chem. Clin. Biochem. 19, 838 (1981); Roche working instructions |
| Bile acids  (TBA) | µmol/l | enzymatic colorimetric determination with 3α-hydroxy-steroid dehydrogenase and NAD | Agape, V. et al., Minerva Dietol Gastroenterol, 35, 159 – 164 (1989); Diazyme working instruction |

### Statistics of clinical pathology

Means, medians and standard deviations of each test group were calculated for several parameters (see tables).

In summary tables of part IB, mean values were rounded, but deviations of means versus control means were calculated with not rounded values. Therefore, slight differences may occur when changes were re-calculated with rounded means. In these tables “deviation vs control” means x-fold of controls expressed as percentages minus 100%.

The following table contains the statistical analyses used in this report. Details were explained in the summary tables in PART IB:

| **Parameter** | **Statistical test** | **Markers in the tables** | **References** |
| --- | --- | --- | --- |
| Blood parameters | For parameters with **bidirectional** changes:  Non-parametric one-way analysis using KRUSKAL-WALLIS test. If the resulting p-value was equal or less than 0.05, a pairwise comparison of each dose group with the control group was performed using WILCOXON-test (two-sided) for the hypothesis of equal medians.  For parameters with **unidirectional** changes:  Pairwise comparison of each dose group with the control group using the WILCOXON-test (one-sided) for the hypothesis of equal medians | * for p < 0.05  ** for p < 0.01 | SIEGEL, S. (1956):  Non-parametric statistics for the behavioural sciences.  McGraw-Hill New York |

## Pathology

### Necropsy

The animals were sacrificed by decapitation under isoflurane anesthesia. The exsanguinated animals were necropsied and assessed by gross pathology.

The female animal No. 390 was sacrificed moribund and was necropsied and assessed by gross pathology as soon as possible after its death.

#

### Organ weights

The following weights were determined in all animals sacrificed on schedule:

1. Anesthetized animals (final body weight)

2. Adrenal glands (fixed)

3. Brain

4. Epididymides

1. Heart
2. Kidneys
3. Liver
4. Ovaries (fixed)
5. Prostate (ventral and dorsolateral part together, fixed)
6. Seminal vesicles with coagulating glands (fixed)
7. Spleen
8. Testes
9. Thymus (fixed)
10. Thyroid glands (with parathyroid glands) (fixed)
11. Uterus with cervix

All paired organs were weighed together (left and right).

### Organ/tissue fixation

The following organs or tissues were fixed in 4% neutral buffered formaldehyde solution or in modified Davidson’s solution:

1. All gross lesions
2. Adrenal glands
3. Aorta
4. Bone marrow (femur)
5. Brain
6. Cecum
7. Cervix
8. Coagulating glands
9. Colon
10. Duodenum
11. Epididymides (modified Davidson’s solution)
12. Esophagus
13. Extraorbital lacrimal glands
14. Eyes with optic nerve (modified Davidson’s solution)
15. Femur with knee joint
16. Harderian glands
17. Heart
18. Ileum
19. Jejunum (with Peyer’s patches)
20. Kidneys
21. Larynx
22. Liver
23. Lungs
24. Lymph nodes (mesenteric and axillary lymph nodes)
25. Mammary gland (male and female)
26. Nose (nasal cavity)
27. Ovaries
28. Oviducts
29. Pancreas
30. Parathyroid glands
31. Pharynx
32. Pituitary gland
33. Prostate
34. Rectum
35. Salivary glands (mandibular and sublingual glands)
36. Sciatic nerve
37. Seminal vesicles
38. Skeletal muscle
39. Skin
40. Spinal cord (cervical, thoracic and lumbar cord)
41. Spleen
42. Sternum with marrow
43. Stomach (forestomach and glandular stomach)
44. Testes (modified Davidson’s solution)
45. Thymus
46. Thyroid glands
47. Trachea
48. Urinary bladder
49. Uterus
50. Vagina

The eyes with optic nerve of the female animal that was sacrificed intercurrently were fixed in 4% neutral buffered formaldehyde solution.

From the liver of all animals sacrificed on schedule, each one slice of the Lobus dexter medialis and the Lobus sinister lateralis was fixed in Carnoy’s solution and embedded in paraplast.

### Sampling of selected tissues for further analyses

For further analysis tissue samples of selected organs were carefully removed and further processed:

1. Kidneys (cranial and caudal end from one kidney)
2. Liver (Lobus caudatus and processus papillaris)

The tissues were sampled in pre-cooled (dry ice) original Eppendorf tubes and snap frozen in liquid nitrogen. The samples were stored at -80°C to be further processed on request of the sponsor.

### Histopathology

The order of test substances listed here is in line with the order of test groups within the different cohorts as performed in the animal experiment.

Fixation was followed by histotechnical processing, examination by light microscopy and assessment of findings according to the table below:

For males and females of the high and low dose level: All observed gross lesions - hematoxylin and eosin (H&E) stain, microscopic examination.

For males and females: High dose - kidney, liver, lungs, ovaries (f), testes (m), thyroid glands: hematoxylin and eosin (H&E) stain, microscopic examination of all animals

Males - Low dose group: Kidneys, liver, thyroid glands - hematoxylin and eosin (H&E) stain, microscopic examination of all animals

Males – Low dose groups: Lungs, testes - H&E stained slides, no microscopic examination

Females - Low dose group: Liver hematoxylin and eosin (H&E) stain, microscopic examination of all animals

Females - Low dose group: Kidneys, lungs, ovaries, thyroid gland: H&E stained slides, no microscopic examination

Immunohistochemistry was performed with Rat alpha 2u-Globulin (AUG) MAb (Clone 129736), Mouse IgG1, purchased from R&D Systems, Catalog # MAB586 as described in Cesta et al. (2013).

The organs were trimmed according to the “Revised guides for organ sampling and trimming in rats and mice” (Ruehl-Fehlert et al., 2003; Kittel et al., 2004; Morawietz et al., 2004).

A correlation between gross lesions and histopathological findings was attempted.

**Peer review**

After completion of the histopathological assessment by the study pathologist an internal peer review was performed by Dr. Maria Cecilia Rey Moreno (BASF SE, Ludwigshafen) including organs, test groups and sexes according to the tables below. Results presented in this report reflect the consensus opinion of the study pathologist and the peer review pathologist.

|  | **Male** | | **Female** | |
| --- | --- | --- | --- | --- |
| **Test substance**  (Test group) | **Low dose** | **High dose** | **Low dose** | **High dose** |
| **Control** | Ki, Li, Lu, Th | | Ki, Li, Th | |
| **DCPD** | Ki, Li, Th | Ki, Li, Th | Li | Li |

Ki = kidneys

Li = liver

Lu = lungs

Th = thyroid

### Statistics of pathology

Means and standard deviations were calculated. In addition, the following statistical analyses were carried out:

| **Parameter** | **Statistical test** | **Markers in the tables** | **References** |
| --- | --- | --- | --- |
| Weight parameters | Non-parametric one-way analysis using KRUSKAL-WALLIS H test (two-sided). If the resulting p-value was equal or less than 0.05, a pairwise comparison of each test group with the control group was performed using WILCOXON-test (two-sided) for the equal medians | * for p ≤ 0.05  ** for p ≤ 0.01 | HETTMANNSPERGER, T.P. (1984): Statistical Inference based on Ranks, John Wiley & Sons New York, 132-140.  International Mathematical and Statistical Libraries, Inc., 2500 Park West Tower One, Houston, Texas 77042-3020, USA, nakl-1 - nakl-3  MILLER, R.G. (1981): Simultaneous Statistical Inference, Springer-Verlag New York Inc., 165-167  NIJENHUIS, A. and S.W. WILF (1978): Combinatorial Algorithms, Academic Press, New York, 32-33 |

**References**

Anderson G.D. (2004) Pharmacogenetics and enzyme induction/inhibition properties of antiepileptic drugs. Neurology. 63 (10 Suppl 4): S3-8

Cesta MF, Hard GC, Boyce JT, Ryan MJ, Chan PC, Sills RC. (2013). Complex histopathologic response in rat kidney to oral β-myrcene: an unusual dose-related nephrosis and low-dose alpha2u-globulin nephropathy. Toxicol Pathol.;41(8):1068-77.

Greaves, P. (2011) Histopathology of Preclinical Toxicity Studies. 4^th^ edition, Academic Press

Hall, A.P., Elcombe, C.R., Foster, J.R., Harada, T., Kaufmann, W., Knippel, A., Küttler, K., Malarkey, D.E., Maronpot, R.R., Nishikawa, A., Nolte, T., Schulte, A., Strauss, V., York, M.J. (2012) Liver Hypertrophy: A Review of Adaptive (Adverse and Non-adverse) Changes—Conclusions from the 3rd International ESTP Expert Workshop, Toxicologic Pathology, 40 (7), 971-994

Kittel B, Ruehl-Fehlert C, Morawietz G, Klapwijk J, Elwell MR, Lenz B, O'Sullivan MG, Roth DR, Wadsworth PF (2004) Revised guides for organ sampling and trimming in rats and mice - Part 2. Exp *Toxicol Pathol* 55: 413–431

Morawietz G, Ruehl-Fehlert C, Kittel B, Bube A, Keane K, Halm S, Heuser A, Hellmann J (2004) Revised guides for organ sampling and trimming in rats and mice - Part 3. *Exp Toxicol Pathol* 55: 433–449

Rosol, T.J., De Lellis, R.A., Harvey, P.W., Sutcliffe, C. (2013). Endocrine System. In: Haschek and Rousseaux’s handbook of toxicologic pathology (W.M. Haschek, C.G. Rousseaux, M.A. Wallig, eds.), 3rd ed., Vol 1, pp 2459-2460. Academic Press.

Ruehl-Fehlert C, Kittel B, Morawietz G, Deslex P, Keenan C, Mahrt CR, Nolte T, Robinson M, Stuart BP, Deschl U (2003) Revised guides for organ sampling and trimming in rats and mice - Part 1. Exp Toxicol Pathol 55: 91–106
