# Supplementary material for: Utility of in vivo metabolomics to support read-across for UVCB substances under REACH
Source: Arch Toxicol. 2024 Jan 24;98(3):755–68. doi: 10.1007/s00204-023-03638-6 (PMC10861390; doi:10.1007/s00204-023-03638-6)
Supplement: Supplementary file 2 — Supplementary file2 (DOCX 32 KB) [file 204_2023_3638_MOESM2_ESM.docx]

Supplementary Material Tox Findings LOA Substances

| **LOA-01** | **Test**  **group** | **Dose**  **[mg/kg bw/d]** | **Males** | **Females** |
| --- | --- | --- | --- | --- |
| **Clinical examinations** | 12 | 1000 | No adverse findings | No adverse findings |
|  | 11 | 300 | No adverse findings | No adverse findings |
| **Clinical pathology** | 12 | 1000 | - Increased red blood cell metabolism (RETA↑) - Hypocoagulability (HQT↑) - Liver cell membrane degradation (ALT↑) | - Hypocoagulability (HQT↑) - Acute phase reaction (WBC↑, LYMPHA↑, MONOA↑) - Liver cell membrane degradation (ALT↑) - Liver cell dysfunction (CHOL↑) - Electrolyte/mineral imbalance (CA↑, INP↑, K↑) |
|  | 11 | 300 | - Hypocoagulability (HQT↑) | No adverse findings |
| **Pathology** | 12 | 1000 | - Liver weight ↑ - Liver cell hypertrophy | - Liver weight ↑ - Liver cell hypertrophy |
|  | 11 | 300 | - Liver cell hypertrophy | No adverse findings |

| **LOA-02** | **Test**  **group** | **Dose**  **[mg/kg bw/d]** | **Males** | **Females** |
| --- | --- | --- | --- | --- |
| **Clinical examinations** | 14 | 750 | - Body weight loss | - Body weight loss |
|  | 13 | 250 | No adverse findings | No adverse findings |
| **Clinical pathology** | 14 | 750 | - Increased red blood cell metabolism (RETA↑) - Acute phase reaction NEUTA↑) - Liver cell membrane degradation (ALT↑) - Liver cell dysfunction (GLUC↓) | - Increased red blood cell metabolism (RETA↑) - Acute phase reaction (NEUTA↑) - Liver cell membrane degradation (ALT↑) - Liver cell dysfunction (CHOL↑, TRIG↑) - Electrolyte/mineral imbalance (INP↑, CA↑, K↑, CL↓) |
|  | 13 | 250 | - Increased red blood cell metabolism (RETA↑)   - Liver cell dysfunction   (GLUC↓) | No adverse findings |
| **Pathology** | 14 | 750 | - Liver weight ↑ - Liver cell hypertrophy | - Liver weight ↑ - Liver cell hypertrophy |
|  | 13 | 250 | - Liver weight ↑ - Liver cell hypertrophy | No adverse findings |

| **LOA-03** | **Test**  **group** | **Dose**  **[mg/kg bw/d]** | **Males** | **Females** |
| --- | --- | --- | --- | --- |
| **Clinical examinations** | 25 | 500 | - Body weight loss | - Body weight loss |
|  | 24 | 150 | No adverse findings | No adverse findings |
| **Clinical pathology** | 25 | 500 | - Increased red blood cell metabolism (RETA↑, TBIL↑) - Hypocoagulability (HQT↑) - Acute phase reaction (WBC↑, LYMPHA↑) - Liver cell membrane degradation (ALT↑) - Liver cell dysfunction (CHOL↑, TPROT↓, ALB↓, TBA↑) | - Regenerative anemia (RETA↑, HGB↓, HCT↓, MCH↓, MCHC↓, TBIL↑) - Hypocoagulability (HQT↑) - Liver cell membrane degradation (ALT↑) - Liver cell dysfunction (CHOL↑, TPROT↓, ALB↓, TRIG↑) - Electrolyte imbalance (NA↓, CL↓) |
|  | 24 | 150 | - Hypocoagulability (HQT↑) - Liver cell membrane degradation (ALT↑) - Liver cell dysfunction (CHOL↑) | - Regenerative anemia (RETA↑, HGB↓, HCT↓, MCH↓) - Liver cell dysfunction (CHOL↑) |
| **Pathology** | 25 | 500 | - Liver weight ↑ - Liver cell hypertrophy prominent nucleoli | - Liver weight ↑ - Liver cell hypertrophy prominent nucleoli |
|  | 24 | 150 | No adverse findings | - Liver cell hypertrophy |

| **LOA-04** | **Test**  **group** | **Dose**  **[mg/kg bw/d]** | **Males** | **Females** |
| --- | --- | --- | --- | --- |
| **Clinical examinations** | 18 | 200 | - Body weight loss | - Body weight loss |
|  | 17 | 70 | No adverse findings | No adverse findings |
| **Clinical pathology** | 18 | 200 | No adverse findings | - Liver cell dysfunction (HQT↓, CHOL↑, TRIG↑) - Electrolyte/mineral imbalance (INP↑, CA↑, CL↓) |
|  | 17 | 70 | No adverse findings | No adverse findings |
| **Pathology** | 18 | 200 | No adverse findings | - Liver weight ↑  - Liver cell hypertrophy |
|  | 17 | 70 | No adverse findings | No adverse findings |

| **LOA-05** | **Test**  **group** | **Dose**  **[mg/kg bw/d]** | **Males** | **Females** |
| --- | --- | --- | --- | --- |
| **Clinical examinations** | 20 | 750 | - Body weight loss | - Body weight loss |
|  | 19 | 250 | No adverse findings | No adverse findings |
| **Clinical pathology** | 20 | 750 | - Increased red blood cell metabolism (RETA↑) - Liver cell membrane degradation (ALT↑) | - Increased red blood cell metabolism (RETA↑) - Acute phase reaction (NEUTA↑, MONOA↑, MONO↑) - Liver cell membrane degradation (ALT↑) - Liver cell dysfunction (GGT↑, TBIL↑, TRIG↑, UREA↓) - Electrolyte imbalance (K↑, Cl↓) |
|  | 19 | 250 | No adverse findings | No adverse findings |
| **Pathology** | 20 | 750 | - Liver weight ↑ - Liver cell hypertrophy | - Liver weight ↑ - Liver cell hypertrophy |
|  | 19 | 250 | No adverse findings | No adverse findings |

| **LOA-06** | **Test**  **group** | **Dose**  **[mg/kg bw/d]** | **Males** | **Females** |
| --- | --- | --- | --- | --- |
| **Clinical examinations** | 22 | 1000 | - Body weight loss | - Body weight loss |
|  | 21 | 300 | No adverse findings | No adverse findings |
| **Clinical pathology** | 22 | 1000 | - Acute phase reaction (NEUTA↑, MONOA↑) - MONO↑, EOS↓ - Liver cell membrane degradation (ALT↑) - Liver cell dysfunction (GGT↑, TBIL↑) | - Increased red blood cell metabolism (RETA↑) - Acute phase reaction (WBC↑, LYMPHA↑, NEUTA↑, MONOA↑) - Liver cell membrane degradation (ALT↑) - Liver cell dysfunction (GGT↑, TBIL↑, TRIG↑, CHOL↑, INP↑) |
|  | 21 | 300 | No adverse findings | - Increased red blood cell metabolism (RETA↑) - Acute phase reaction (WBC↑, LYMPHA↑) |
| **Pathology** | 22 | 1000 | - Liver weight ↑ - Liver cell hypertrophy | - Liver weight ↑ - Liver cell hypertrophy |
|  | 21 | 300 | No adverse findings | No adverse findings |

| **LOA-07** | **Test**  **group** | **Dose**  **[mg/kg bw/d]** | **Males** | **Females** |
| --- | --- | --- | --- | --- |
| **Clinical examinations** | 16 | 600 | - Body weight loss | - Body weight loss |
|  | 15 | 200 | No adverse findings | No adverse findings |
| **Clinical pathology** | 16 | 600 | - Increased red blood cell metabolism (RETA↑) - Acute phase reaction? (LYMPH↓, MONOA↑) - MONO↑, NEUT↑, NEUTA↑, - PLT↓ - Liver cell membrane degradation (ALT↑) | - Increased red blood cell metabolism (RETA↑) - Liver cell membrane degradation (ALT↑) - Liver cell dysfunction (HQT↓, GLUC↓, CHOL↑, TRIG↑) - Electrolyte/mineral imbalance (INP↑, CA↑, CL↓, K↑) |
|  | 15 | 200 | - Liver cell membrane degradation (ALT↑) | No adverse findings |
| **Pathology** | 16 | 600 | - Liver weight ↑ - Liver cell hypertrophy | - Liver weight ↑ - Liver cell hypertrophy |
|  | 15 | 200 | - Liver weight ­ | No adverse findings |

| **LOA-08** | **Test**  **group** | **Dose**  **[mg/kg bw/d]** | **Males** | **Females** |
| --- | --- | --- | --- | --- |
| **Clinical examinations** | 27 | 300 | - Body weight loss | - Body weight loss |
|  | 26 | 100 | No adverse findings | No adverse findings |
| **Clinical pathology** | 27 | 300 | - Liver cell dysfunction (HQT↓, CHOL↑, TRIG↑) - Electrolyte/mineral imbalance (INP↑, CL↓) | - Liver cell dysfunction (CHOL↑, TRIG↑) - Increased red blood cell metabolism (RETA↑) - Electrolyte/mineral imbalance (INP↑, NA↓, CL↓) |
|  | 26 | 100 | No adverse findings | No adverse findings |
| **Pathology** | 27 | 300 | - Liver weight ↑ - Liver cell hypertrophy | - Liver weight ↑ - Liver cell hypertrophy |
|  | 26 | 100 | No adverse findings | No adverse findings |

| **LOA-09** | **Test**  **group** | **Dose**  **[mg/kg bw/d]** | **Males** | **Females** |
| --- | --- | --- | --- | --- |
| **Clinical examinations** | 29 | 200 | No adverse findings | - Body weight loss |
|  | 28 | 70 | No adverse findings | No adverse findings |
| **Clinical pathology** | 29 | 200 | - Liver cell dysfunction (CHOL↑, TRIG↑, GLUC↓) - Electrolyte/mineral imbalance (INP↑, CL↓) | - Acute phase reaction (WBC↑, NEUTA↑, LYMPHA↑) - Liver cell dysfunction (CHOL↑, TRIG↑) |
|  | 28 | 70 | No adverse findings | No adverse findings |
| **Pathology** | 29 | 200 | - Liver weight ↑ - Liver cell hypertrophy and single cell necrosis | - Liver weight ↑ |
|  | 28 | 70 | No adverse findings | No adverse findings |

| **LOA-10** | **Test**  **group** | **Dose**  **[mg/kg bw/d]** | **Males** | **Females** |
| --- | --- | --- | --- | --- |
| **Clinical examinations** | 31 | 1000 | No adverse findings | - Body weight loss |
|  | 30 | 300 | No adverse findings | No adverse findings |
| **Clinical pathology** | 31 | 1000 | - Increased red blood cell metabolism (RETA↑) - Hypocoagulability (HQT↑) | No adverse findings |
|  | 30 | 300 | No adverse findings | No adverse findings |
| **Pathology** | 31 | 1000 | No adverse findings | No adverse findings |
|  | 30 | 300 | No adverse findings | No adverse findings |

| **LOA-11** | **Test**  **group** | **Dose**  **[mg/kg bw/d]** | **Males** | **Females** |
| --- | --- | --- | --- | --- |
| **Clinical examinations** | 33 | 500 | - Body weight loss | No adverse findings |
|  | 32 | 150 | No adverse findings | No adverse findings |
| **Clinical pathology** | 33 | 500 | - (regenerative) Anemia (HGB↓, MCHC↓, RETA↑, TBIL↑) - Hypocoagulability (HQT↑) - Liver cell dysfunction (TPROT↓, ALB↓) | - Regenerative anemia (HGB↓, MCHC↓, RETA↑, TBIL↑) - Liver cell dysfunction (TPROT↓, ALB↓) |
|  | 32 | 150 | No adverse findings | - Regenerative anemia (HGB¯, RETA­) |
| **Pathology** | 33 | 500 | - Liver weight ↑ - Liver cell hypertrophy and prominent nucleoli | - Liver weight ↑ - Liver cell hypertrophy and prominent nucleoli |
|  | 32 | 150 | No adverse findings | No adverse findings |

| **LOA-12** | **Test**  **group** | **Dose**  **[mg/kg bw/d]** | **Males** | **Females** |
| --- | --- | --- | --- | --- |
| **Clinical examinations** | 35 | 1000 | - Body weight loss | No adverse findings |
|  | 34 | 300 | No adverse findings | No adverse findings |
| **Clinical pathology** | 35 | 1000 | - Increased red blood cell metabolism (RETA↑) - Liver cell dysfunction (GGT↑, GLUC↓) | - Regenerative anemia (RETA↑, HGB↓, HCT↓, MCHC↓) - Liver cell dysfunction (GGT↑, TRIG↑) - Electrolyte imbalance (NA↓, CL↓) |
|  | 34 | 300 | No adverse findings | - (regenerative) Anemia (HGB↓, HCT↓) |
| **Pathology** | 35 | 1000 | - Liver weight ↑ - Liver cell hypertrophy and prominent nucleoli | - Liver weight ↑ - Liver cell hypertrophy and prominent nucleoli |
|  | 34 | 300 | No adverse findings | No adverse findings |

| **BASF LOA-1** | **Test**  **group** | **Dose**  **[mg/kg bw/d]** | **Males** | **Females** |
| --- | --- | --- | --- | --- |
| **Clinical examinations** | 12 | 1000 | - Apathy, piloerection and semi-closed eyelids during the first days of application - Body weight loss on study day 3 and 13 - Decreased body weight and body weight change on study days 3 to 13 | - Apathy, piloerection and semi-closed eyelids during the first days of application - Body weight loss on study day 3 |
|  | 11 | 300 | - Apathy, piloerection and semi-closed eyelids during the first days of application | - Semi-closed eyelids during the first days of application |
| **Clinical Pathology** | 12 | 1000 | - Increased red blood cell metabolism (RETA↑, TBIL↑) - Hypocoagulability (HQT↑) - Liver cell membrane degradation (ALT↑) - Liver cell dysfunction (CHOL↑, UREA↓, GLUC↓, TRIG↓) | - Regenerative anemia (RBC↓, HGB↓, HCT↓, MCHC↓, RETA↑) - Hypocoagulability (HQT↑) - Liver cell membrane degradation (ALT↑) - Liver cell dysfunction (CHOL↑, TRIG↑) |
|  | 11 | 300 | - Hypocoagulability (HQT↑) | - Regenerative anemia (HGB↓, HCT↓, RETA↑) - Hypocoagulability (HQT↑) - Liver cell membrane degradation (ALT↑) - Liver cell dysfunction (CHOL↑, TRIG↑) |
| **Pathology** | 12 | 1000 | - Final body weight ↓ | - Liver weight ↑  - Liver cell hypertrophy |
|  | 11 | 300 | No adverse finding | - Liver weight ↑  - Liver cell hypertrophy |

| **BASF LOA-2** | **Test**  **group** | **Dose**  **[mg/kg bw/d]** | **Males** | **Females** |
| --- | --- | --- | --- | --- |
| **Clinical Examinations** | 14 | 1000 | - Apathy, piloerection and semi-closed eyelids during the first days of application - Body weight loss on study day 3 and 13 - Decreased body weight and body weight change on study days 3 to 13 | - Piloerection and semi-closed eyelids during the first days of application - Slight body weight loss on study day 3 |
|  | 13 | 300 | Apathy and semi-closed eyelids during the first days of application | Apathy, piloerection and semi-closed eyelids during the first days of application |
| **Clinical Pathology** | 14 | 1000 | - Regenerative anemia (HGB↓, MCHC↓, RETA↑, TBIL↑) - Hypocoagulability (HQT↑) - Liver cell membrane degradation (ALT↑)   Liver cell dysfunction (CHOL↑, TRIG↓) | - Regenerative anemia (HGB↓, RBC↓, HCT↓, MCHC↓, MCV↑, RETA ↑, TBIL ↑) - Liver cell membrane degradation (ALT↑)   - Liver cell dysfunction (CHOL↑, UREA↑, TRIG↑) |
|  | 13 | 300 | - Increased red blood cell metabolism (RETA↑) | - Increased red blood cell metabolism (RETA↑) |
| **Pathology** | 14 | 1000 | - Final body weight ↓ | - Liver weight ↑  - Liver cell hypertrophy |
|  | 13 | 300 | No adverse finding | No adverse finding |

| **BASF LOA-3** | **Test**  **group** | **Dose**  **[mg/kg bw/d]** | **Males** | **Females** |
| --- | --- | --- | --- | --- |
| **Clinical examinations** | 16 | 1000 | - Apathy, piloerection and semi-closed eyelids during the first days of application - Reduced food consumption during the first days of application - Body weight loss on study day 3 and 13 - Decreased body weight and body weight change on study days 3 to 13 | - Apathy, piloerection, unsteady gait, ataxia and semi-closed eyelids during the first days of application - Reduced food consumption during the first days of application - Body weight loss on study day 3 |
|  | 15 | 300 | - Semi-closed eyelids during the first days of application - Reduced food consumption during the first days of application - Decreased body weight and body weight change on study days 3 to 13 | - Apathy, piloerection and semi-closed eyelids during the first days of application - Reduced food consumption during the first days of application - Decreased body weight and body weight change on study days 3 to 10 |
| **Clinical Pathology** | 16 | 1000 | - Increased red blood cell metabolism (RETA↑, TBIL↑) - Hypocoagulability (HQT↑) - Liver cell membrane degradation (ALT↑) - Liver cell dysfunction (CHOL↑) | - Regenerative anemia (RBC↓, HGB↓, MCHC↓, MCV↑, RETA↑, TBIL↑) - Liver cell membrane degradation (ALT↑) - Liver cell dysfunction (CHOL↑, TRIG↑) |
|  | 15 | 300 | - Increased red blood cell metabolism (RETA↑) - Hypocoagulability (HQT↑) | - Increased red blood cell metabolism (RETA↑) |
| **Pathology** | 16 | 1000 | - Final body weight ↓   - Liver weight ↑ | - Liver weight ↑ - Centrilobular liver cell hypertrophy |
|  | 15 | 300 | No adverse finding | No adverse findings |
