# Supplementary material for: Utility of in vivo metabolomics to support read-across for UVCB substances under REACH
Source: Arch Toxicol. 2024 Jan 24;98(3):755–68. doi: 10.1007/s00204-023-03638-6 (PMC10861390; doi:10.1007/s00204-023-03638-6)
Supplement: Supplementary file 3 — Supplementary file3 (DOCX 21 KB) [file 204_2023_3638_MOESM3_ESM.docx]

Supplementary Material Tox Findings Marker Substances

| **Indene** | **Test**  **group** | **Dose**  **[mg/kg bw/d]** | **Males** | **Females** |
| --- | --- | --- | --- | --- |
| **Clinical examinations** | 48 | 450 | - Body weight loss on study day 3 - Decreased body weight gain from study  day 0 to 13 - Decreased body weight on study days 3 to 13 | - Body weight loss on study day 3 |
|  | 47 | 100 | No adverse finding | No adverse finding |
| **Clinical pathology** | 48 | 450 | - Regenerative anemia (RBC↓, HGB↓, HCT↓, RETA↑, MCHC↓, TBIL↑) - Liver cell membrane degradation (ALT↑, AST↑) - Liver cell dysfunction - (TPROT↓, GLOB↓, TRIG↓) | - Regenerative anemia (RBC↓, HGB↓, MCHC↓, RETA↑, MCV↑, TBIL↑) - Stress (EOSA↓) - Liver cell dysfunction (TPROT↓, GLOB↓) |
|  | 47 | 100 | No adverse finding | No adverse finding |
| **Pathology** | 48 | 450 | No adverse finding | - Liver weight females ↑ |
|  | 47 | 100 | No adverse finding | No adverse finding |

| **Cyclopentane** | **Test**  **group** | **Dose**  **[mg/kg bw/d]** | **Males** | **Females** |
| --- | --- | --- | --- | --- |
| **Clinical examinations** | 46 | 1000 | No adverse finding | No adverse finding |
|  | 45 | 300 | No adverse finding | No adverse finding |
| **Clinical pathology** | 46 | 1000 | No adverse finding | No adverse finding |
|  | 45 | 300 | No adverse finding | No adverse finding |
| **Pathology** | 46 | 1000 | No adverse finding | No adverse finding |
|  | 45 | 300 | No adverse finding | No adverse finding |

| **Dicyclopentandiene** | **Test**  **group** | **Dose**  **[mg/kg bw/d]** | **Males** | **Females** |
| --- | --- | --- | --- | --- |
| **Clinical examinations** | 44 | 150 | - Body weight loss on study day 3 | - Body weight loss on study day 3 |
|  | 43 | 50 | - No adverse finding | - Body weight loss on study day 3 |
| **Clinical pathology** | 44 | 150 | - Liver cell dysfunction (CHOL↑, HQT↓, GLUC↓) | - Liver cell dysfunction (CHOL↑, TPROT↓, GLOB↓, AST↑) - Bone/acidosis (ALP↑, INP↑) |
|  | 43 | 50 | No adverse finding | No adverse finding |
| **Pathology** | 44 | 150 | No adverse finding | - Centrilobular liver cell hypertrophy - Increased single cell necrosis in the liver |
|  | 43 | 50 | No adverse finding | No adverse finding |

| **Naphthalene** | **Test**  **group** | **Dose**  **[mg/kg bw/d]** | **Males** | **Females** |
| --- | --- | --- | --- | --- |
| **Clinical examinations** | 42  (50 (only ♀) | 600 | - Body weight loss on study day 3 - Decreased body weight gain on study day 13 and from study day 0 to 13 | - Body weight loss on study day 3 and 7 - Decreased body weight gain from study  day 0 to 13 - 1 female moribund - Piloerection in female animals - Poor general condition in female animals - Reduced nutritional condition in female animals - Pale skin in female animals - High stepping gait in female animals |
|  | 41 | 250 | - Body weight loss on study day 3 - Decreased body weight gain from study  day 0 to 13 | No adverse finding |
| **Clinical pathology** | 42  (50 (only ♀) | 600 | - Increased red blood cell metabolism (RETA↑) - Liver cell membrane degradation (ALT↑)   - Liver cell dysfunction (TRIG↓, TBIL↑) | - Increased red blood cell metabolism (RETA↑) - Acute phase reaction (MONOA↑) - Liver cell membrane degradation (ALT↑) - Liver cell dysfunction (GLUC↑, CHOL↑) |
|  | 41 | 250 | No adverse finding | No adverse finding |
| **Pathology** | 42  (50 (only ♀) | 600 | - Prominent nucleoli in liver cells ↑ | - One animal sacrificed moribund with degeneration in kidney - Liver weight ↑ - Liver cell hypertrophy |
|  | 41 | 250 | No adverse finding | No adverse finding |

| **Xylene** | **Test**  **group** | **Dose**  **[mg/kg bw/d]** | **Males** | **Females** |
| --- | --- | --- | --- | --- |
| **Clinical examinations** | 40 | 1000 | No adverse findings | No adverse findings |
|  | 39 | 300 | No adverse findings | No adverse findings |
| **Clinical pathology** | 40 | 1000 | - Liver cell dysfunction (HQT↓, CHOL↑) | - Liver cell dysfunction (HQT↓, CHOL↑) |
|  | 39 | 300 | No adverse findings | No adverse findings |
| **Pathology** | 40 | 1000 | - Liver weight ↑  - Liver cell hypertrophy | - Liver weight ↑  - Liver cell hypertrophy |
|  | 39 | 300 | No adverse findings | No adverse findings |

| **Benzene** | **Test**  **group** | **Dose**  **[mg/kg bw/d]** | **Males** | **Females** |
| --- | --- | --- | --- | --- |
| **Clinical examinations** | 38 | 1000 | - Hyperexcitability and twitching - Reduced food consumption - Reduced body weight and body weight gain | - Hyperexcitability |
|  | 37 | 300 | No adverse findings | No adverse findings |
| **Clinical pathology** | 38 | 1000 | - Lymphopenia (WBC↓, LYMPHA↓, EOSA↓) - MONO↑ | - Lymphopenia (WBC↓, LYMPHA↓, EOSA↓) - MONO↑ |
|  | 37 | 300 | - Lymphopenia (WBC↓, LYMPHA↓, EOSA↓) - MONO↑ | - Lymphopenia (WBC¯, LYMPHA↓, EOSA↓) - MONO↑ |
| **Pathology** | 38 | 1000 | No adverse findings | - Single cell necrosis in liver |
|  | 37 | 300 | No adverse findings | - Single cell necrosis in liver |
