# Supplementary material for: Utility of in vivo metabolomics to support read-across for UVCB substances under REACH
Source: Arch Toxicol. 2024 Jan 24;98(3):755–68. doi: 10.1007/s00204-023-03638-6 (PMC10861390; doi:10.1007/s00204-023-03638-6)
Supplement: Supplementary file 5 — Supplementary file5 (PPTX 125 KB) [file 204_2023_3638_MOESM5_ESM.pptx]

## Slide 1
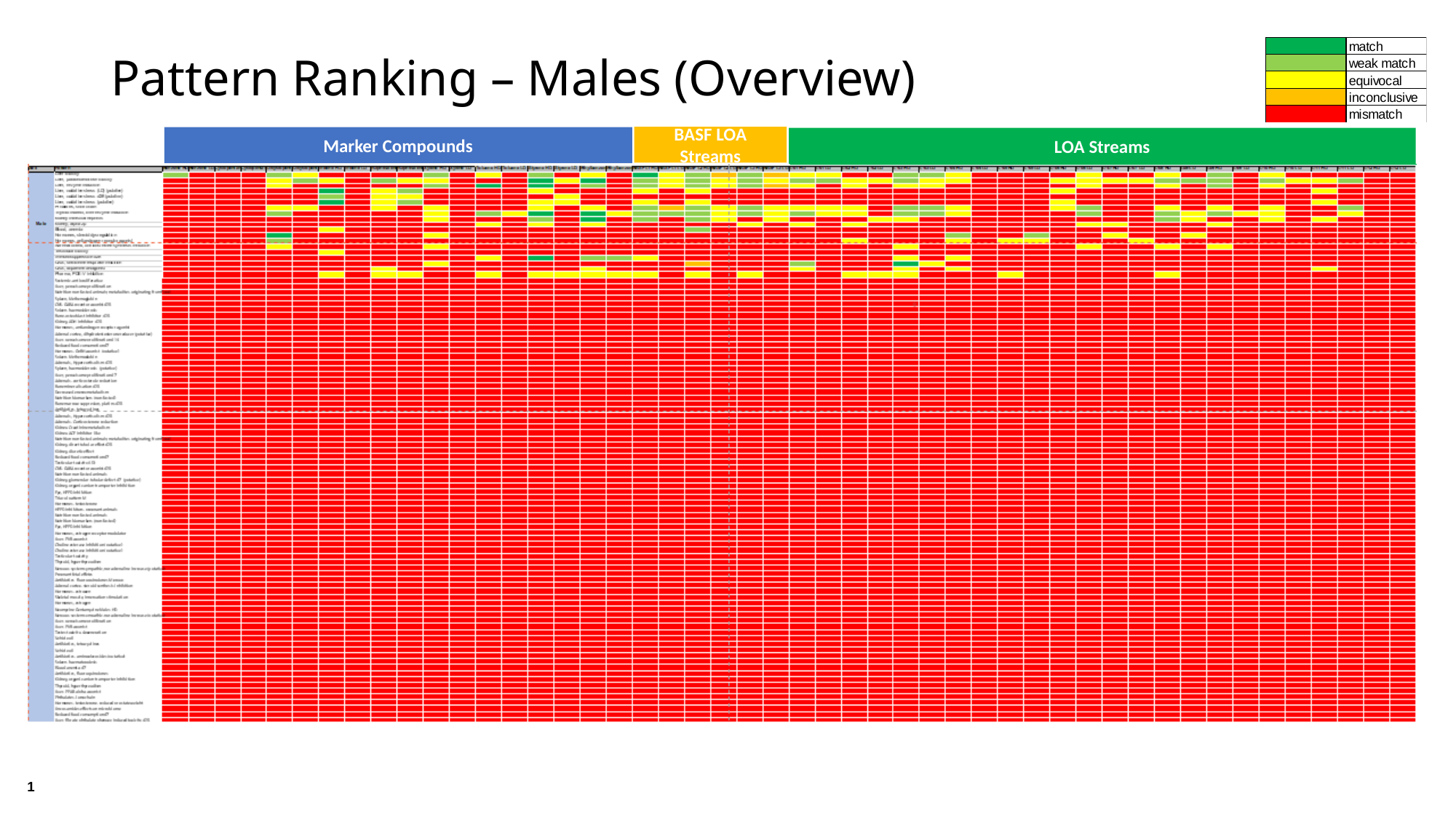

# Pattern Ranking – Males (Overview)
BASF LOA Streams
Marker Compounds
LOA Streams
1

## Slide 2
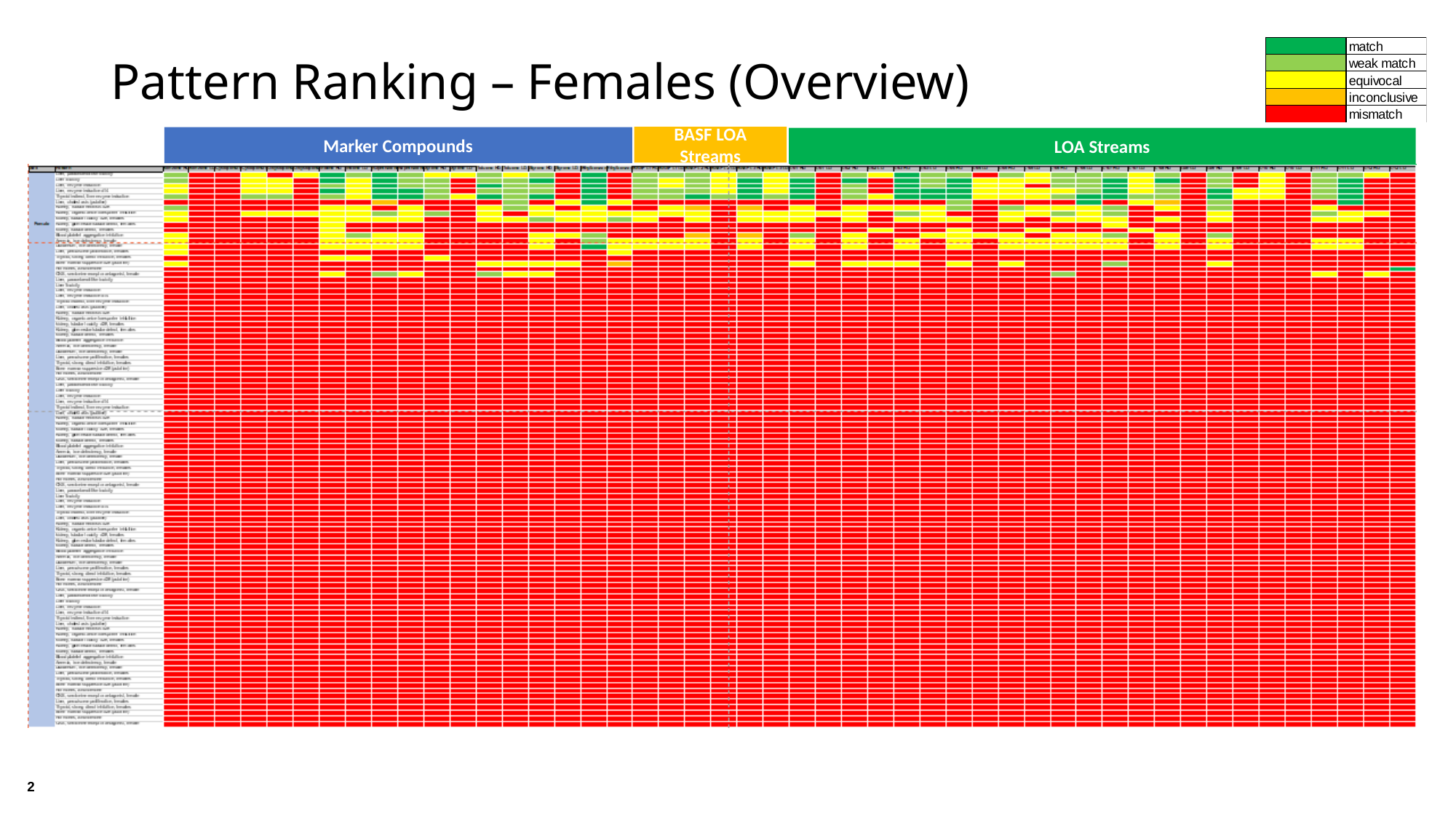

# Pattern Ranking – Females (Overview)
BASF LOA Streams
Marker Compounds
LOA Streams
2
